# Agent-based persuasion model with concessions dependent on emotion and time beliefs

**DOI:** 10.1371/journal.pone.0333078

**Published:** 2025-09-25

**Authors:** Jinghua Wu, Ruiyang Cao, Ya Zhang, Yan Li

**Affiliations:** School of management, China University of Mining and Technology - Beijing, Beijing, China; University of Malta, MALTA

## Abstract

Automated negotiation agents require human-like adaptability in emotionally charged and time-constrained settings. This study introduces an Emotion-Time Dual-Process Framework that integrates the Appraisal Tendency Framework with dynamic temporal modeling. Emotions are decomposed into pleasantness and certainty dimensions and mapped to six emotional persuasion strategies. A variable-rate time function is designed to capture the perceptions of dynamic time pressure. Emotion and time pressure jointly drive a state-dependent concession updating model. The proposed framework was validated through a series of simulation experiments based on different scenarios. The results demonstrate that the proposed framework has significant advantages in improving negotiation success rates, joint utility, and outcome fairness against baseline models. In particular, incorporating emotional factors reduces utility disparity between parties by 28.55%, while the proposed time function improves negotiation efficiency by 12.99% without sacrificing fairness or the success rate. This study provides a theorical basis for developing highly more human-like and adaptive intelligent negotiation systems.

## 1. Introduction

With the rapid advancement of artificial intelligence (AI), agent-based automated negotiation systems have shown considerable potential across various business sectors [1,2]. AI-powered negotiating agents are designed to emulate human behaviors such as autonomy, initiative, collaboration, and dynamic adjustment, enabling them to autonomously perform predefined negotiation tasks in response to changes in the external environment [3,4]. Despite significant progress in existing research, challenges remain in enhancing the anthropomorphism of agents and the sophistication of their decision-making processes [5,6]. Future optimization efforts should focus on developing intelligent software that exhibits with negotiation capabilities comparable with humans to facilitate effective interactions. Considering that emotions are a critical attribute unique to humans and natural human-machine interactions can benefit from agents exhibiting emotional behavior [7,8], it is essential to incorporate emotional elements into agent-based persuasion [9]. Moreover, most human interactions are time-dependent, and individual attitudes toward time significantly impact the formulation of concessions that address negotiation time pressures [10–12]. Therefore, this research aims to enhance the modeling of the persuasive behavior of an agent in automated negotiation by emphasizing emotion and timing.

Recent research has focused on modeling agents to reflect fundamental human mental states, by focusing on emotion [13,14]. Introducing emotions into agent-based persuasion is crucial, because emotions play a vital role in human interactions. Similarly, users have anthropomorphic expectations and requirements from agents during human–agent interactions [15]. Incorporating emotional components facilitates natural and effective interactions between agents and humans. Although agents lack emotions, they can be designed for specific roles. Defining role orientations and basic capabilities enables agents to exhibit emotional feedback behavior that enhances their personification as service-oriented entities. However, it is imperative to note that negotiation, similar to multi-round game interaction behavior, is inherently a multi-dimensional cognitive decision-making process. Therefore, negotiating agents should assess the benefits of current offers and engage in complex cognitive processes [16,17], such as risk prediction, attribution of responsibility for the intentions of the adversary, and the ongoing regulation of their sense of control in a dynamic environment. Existing studies [8] have demonstrated that human behavior results from the interplay between cognitive and emotional functions, and agents can generate and analyze emotions and their effects by simulating the human thinking mechanism, thereby accurately modeling human behavioral patterns driven by the interplay of cognition and emotion.

Considerable research has yielded valuable insights into the impact of emotions on decision making [18–22], with current studies primarily grounded in the concepts of valence-based [23] and appraisal tendency framework (ATF) theories [24]. Research based on emotional valence generally categorizes emotions as positive or negative [7,9], assuming that emotions with the same valence exert identical effects on decision-making processes. Specifically, positive emotions might lead individuals to make optimistic judgments, while negative emotions might induce pessimistic judgments. In contrast, the ATF emphasizes the relationship between emotions and cognitive processes. Based on this framework, emotional experience shapes the cognitive tendencies of an individual and influences the evaluation of the event through the core dimensions of appraisal associated with emotion, ultimately affecting decision-making behavior [25,26]. The ATF distinguishes between the heterogeneity of emotions within the same valence category by analyzing the cognitive evaluative dimensions, such as pleasantness, certainty, and control. While anxiety and anger are classified as negative emotions, anger is associated with high certainty, whereas anxiety is linked to low certainty [27]. Anger might result in external attributions of blame, potentially resulting in risk-taking behavior, while anxiety tends to evoke internal attributions of self-blame, prompting individuals to seek corrective action.

In emotionally charged negotiation scenarios, participants need to constantly balance factors such as pressure, expectations, risk perception, and responsibility attribution. Hence, the interaction between emotions and cognitive processes becomes especially significant [18,28]. While valence-based theories can explain the presence or absence of emotional effects, they fail to address the cognitive mechanisms underlying emotional influence, making it difficult to fully elucidate how emotions contribute to the development of negotiation strategies. In contrast, the ATF offers a dual advantage: it indicates whether emotions affect negotiation and elucidates the underlying mechanisms through which emotions influence negotiation behavior via cognitive dimensions. Consequently, the ATF provides a comprehensive theoretical framework and serves as a foundation for developing emotion-driven, human-like negotiation agents. Consequently, this study adopts the ATF as a theoretical framework to analyze the effect of real-time emotions on the persuasive strategy selection and negotiation behavior of an agent.

The modeling of concessions that can adapt to negotiation time constraints is a significant challenge in automated persuasion research [10,29,30]. Human interactions require time. During the negotiation process, both parties typically operate under time limitations; however, their cognition and understanding of negotiation time can differ markedly [11,12]. This perception of negotiation time can be conceptualized as an internal drive that influences the ability of individuals to regulate the pace of negotiations. For instance, a confident or patient negotiator might exhibit greater certainty and motivation to reach an agreement as the negotiation period progresses, whereas a less confident or impatient participant might prefer to finalize an agreement in a short period. In brief, time pressure exerts a universal influence, consistently motivating negotiators to achieve their objectives. Consequently, it is crucial to consider the attitude of the agent toward time as a key dimension of persuasion. Thus, we further extended the modeling of negotiating agents and improved the dynamic concession updating process of an agent by incorporating its time-related attitudes with the effects of fluctuations in the emotional state of the opponent.

Given the significant roles emotions and time attitudes play in automated negotiation, this paper addresses the following research questions: How can the cognitive evaluation dimensions of immediate emotions be effectively modeled based on ATF, and how can the impact of emotions on decision-making be analyzed accordingly? Furthermore, how can an agent integrate its emotional state and time beliefs to dynamically adjust its concession behavior across multiple rounds of negotiation, thereby enhancing both negotiation efficiency and outcome quality? To address these questions, we propose an agent-based persuasion model where concessions are influenced by emotional states and time beliefs. In summary, this study makes the following contributions to agent-based automated negotiation:

(1) This study proposes a novel Emotion-Time Dual Driven Framework that advances automated negotiation by integrating two critical aspects of human negotiation behavior: emotional dynamics and temporal reasoning. The framework uniquely combines emotional appraisal mechanisms (based on the Appraisal Tendency Framework) with temporal belief modeling to guide negotiation strategies. Unlike existing approaches that consider either emotional factors or time pressure in isolation, our framework enables agents to simultaneously process emotional states and temporal beliefs in each negotiation round, leading to more sophisticated strategy adaptation. This integration provides agents with the capability to balance emotional influences with temporal constraints, more closely approximating human decision-making processes in real-world negotiations. The framework’s dual-input architecture establishes a foundation for developing more advanced negotiation agents that can exhibit both emotional intelligence and temporal awareness.
(2) This study advances the state of automated negotiation by introducing a sophisticated emotion modeling approach based on the Appraisal Tendency Framework (ATF). Moving beyond traditional valence-based approaches that simply categorize emotions as positive or negative, this paper decomposes immediate emotions into two core cognitive dimensions—pleasantness and certainty. This dimensional decomposition enables agents to distinguish between emotions that share the same valence but differ in their cognitive implications. By establishing a systematic mapping from emotional states to cognitive appraisal patterns, we provide agents with a more nuanced understanding of emotional influences on negotiation behavior, significantly enhancing their ability to generate psychologically-grounded responses. This approach effectively integrates psychological theory with computational modeling, significantly enhancing the interpretability, adaptability, and human-likeness of negotiation agents in multi-round dynamic interactions.
(3) This paper introduces a novel approach to temporal modeling in automated negotiation through a nonlinear variable-rate time function. Unlike traditional linear time models that are highly predictable and susceptible to exploitation, our approach captures the dynamic nature of perceived time pressure during negotiations. The variable-rate function enables agents to adjust their temporal reasoning based on negotiation progress, deadline proximity, and strategic considerations. This advancement addresses a significant limitation in existing negotiation systems, where rigid time models fail to reflect the complex temporal dynamics observed in human negotiations. Our model provides agents with more flexible and realistic temporal adaptation capabilities, contributing to more robust negotiation outcomes.

The rest of the paper is organized as follows: Section 2 describes a related theoretical background and gives a summary of the related work. Section 3 presents a description of the proposed model. Section 4 gives a model analysis. Numerical experiments are conducted in Section 5, which also reports the results of sensitive and comparative analyses. Section 6 discusses the theoretical implications, practical implications, and limitations. This paper is concluded in Section 7.

## 2. Theoretical background and related literature

### 2.1 Automated negotiation

Negotiation refers to a communication process between two or more parties aimed at reaching an agreement on a specific issue. Automated negotiation, on the other hand, involves the use of artificial intelligence agent mechanisms to automate this process [31,32]. The negotiation process is inherently complex, requiring consideration of various factors, such as the negotiation protocols [33] and time constraints [10]. Given the broad range of application scenarios for automated negotiation, the design of corresponding models incorporates a wide array of negotiation theories and technical approaches. Despite the diversity of research content, the primary issues in current studies can be categorized into three main areas [34]: (1) negotiation protocol, which defines the set of rules governing agent interactions; (2) negotiation issue, which encompasses the range of subjects on which the negotiating parties must reach an agreement; and (3) concession decision-making model, which refers to the model employed by agents to make dynamic concessions in accordance with the negotiation protocol and issue.

The relative importance of the three primary research questions mentioned above varies depending on the nature of the negotiation and the context in which it occurs. However, the agent’s concession decision-making model remains the central focus of research in this field. In decision modeling studies, the negotiation agreement does not prescribe an optimal strategy for the agent; rather, the agent must independently determine a decision framework and implement it using embedded decision-making methods. Based on the timeline of its development, current automatic negotiation models can be classified into four categories: game theory-based [35], argumentation-based [36], heuristic-based [37], and persuasion-based [38]. In recent years, with advancements in artificial intelligence technology and the increasing depth of related research, agent-based emotional persuasion [16] has emerged as a growing trend in automated negotiation studies.

At this stage, researchers are focused on developing more flexible and diverse emotional persuasion agent models to provide users with a more personalized, engaging, and efficient negotiation experience. However, a key challenge in this process is overcoming the limitations of current research to enable agents to more accurately simulate human emotions and emotion-driven behavioral strategies. This capability is essential for advancing the field of intelligent negotiation and is a primary focus of this study.

### 2.2 Decision theory in automated negotiation

Agent-based automated negotiation primarily involves programmed decision-making through the establishment of negotiation rules, strategies, and objectives. As such, decision theory occupies an important role in the study of automated negotiation, offering valuable frameworks for intelligent agents to make optimal decisions in complex, dynamic, and information-limited environments. This research mainly focuses on three key decision theories: utility theory, multi-attribute decision theory, and behavioral decision theory.

Utility theory forms the foundation of automated negotiation [39]. Its central concept is that an individual’s decision-making preferences can be represented by a utility function, which assigns numerical values to various outcomes, thereby enabling the quantification of the relative value of different options. The utility function is the primary tool within utility theory, used to quantify and analyze the preferences of individuals when confronted with a set of alternatives, thus allowing negotiation agents to make informed decisions.

Multi-attribute decision theory addresses the challenge of trade-offs in multi-objective conflicts encountered in automated negotiations [40]. The theory asserts that individuals evaluate different attributes when making decisions, assessing the utility of each option based on the relative significance of those attributes. This theory has garnered considerable attention due to its ability to provide a comprehensive framework for understanding human decision-making processes, which can help explain why individuals incorporate multiple factors in their decision-making and offer practical insights for addressing complex decision-making problems in real-world scenarios.

Behavioral decision theory challenges traditional assumptions of rationality by integrating psychological bias models, such as the anchoring effect and loss aversion, to enhance the adaptability of human-computer interactions in automated negotiation systems. For instance, human negotiators are often influenced by initial offers, referred to as the anchor point, and prospect theory further quantifies the concept of “loss aversion,” explaining why negotiators exhibit reluctance to accept offers that fall below their psychological reference point. Moreover, emotion, as a complex psychological phenomenon, has long been acknowledged for its irreplaceable role in elucidating human behavioral motives and decision-making processes [28]. Consequently, this decision-making framework is particularly significant in contexts like customer service negotiations, where simulating human behavior is essential.

In summary, research on automated negotiation typically requires the integration of various decision-making theories. The selection of appropriate theories for practical applications depends on the specific negotiation scenario. For instance, competitive negotiations often emphasize game theory, while collaborative negotiations, such as those explored in this study, usually rely more on theories like utility theory, multi-attribute decision theory, and behavioral decision theory. These theories serve as tactical tools to quantify interests, manage trade-offs, and correct biases.

### 2.3 Emotional persuasion

Emotional persuasion is a development arising from traditional automated negotiation, integrating theories such as emotion, persuasion psychology, and belief modification into the negotiation process. This research is based on a non-completely competitive negotiation, where buyers and sellers do not have a completely adversarial relationship in the negotiation process. Instead, they demonstrate a certain willingness to cooperate and negotiate with the desire to achieve mutual benefit. The focus of this research is to create a persuasion pattern by equipping the agent with human attributes. This enables the negotiation parties to better understand the implications of the information obtained during the interaction and complements rationality to achieve strategic effects. Related studies have primarily focused on examining the critical role of emotions in negotiation processes or investigating the effects of different emotional states on strategic choices by modeling the emotions of agents [21,22,41].

The immediate emotions frequently examined in emotional persuasion research include joy, happiness, gratification, anticipation, anxiety, disappointment, sadness, and anger [7,9,20]. These emotions are recognized as key factors influencing agent behavior during negotiations. For instance, Raghunathan et al. [42] discussed how emotional states of similar valence can have distinct but predictable effects on decision-making. Specifically, sadness tends to drive individuals toward high-risk, high-reward options, whereas anxiety typically leads to the selection of low-risk, low-reward alternatives. In contrast, Van Kleef et al. [43] investigated the interpersonal effects of anger and happiness in negotiations through three experiments. Their findings revealed a significant interaction between emotional experience and emotional expression, which in turn influenced the behavior of opponents. Furthermore, an empirical study by Zeelenberg et al. [25] found that specific emotions, such as disappointment and regret, have a direct impact on individuals’ behavioral responses (e.g., complaints or grievances). While these studies offer valuable empirical support for the role of emotions in automated negotiation, the majority of current research still focuses on theoretical assumptions and experimental analyses, and there are still significant shortcomings in the design of agents and implementation of strategies for emotional influence. In particular, further theoretical advancement and model refinement are urgently needed in terms of how to transform specific emotions effectively into computable mechanisms of agent behavior.

As research in emotional persuasion progresses, Wu et al. [7] began exploring the impact of extreme emotions (e.g., happiness vs. anger) in the modeling of emotionally persuasive agents, building on prior empirical studies. They argued that happiness typically encourages agents to adopt positive and cooperative strategies, whereas anger tends to provoke more aggressive or defensive approaches. Building on this foundation, Wang et al. [9] further expanded the emotion categories to include a range of moderate-intensity emotions such as hope, gratitude, and disappointment, and developed models that guide agents’ decision-making based on the intensity of these emotions. However, these studies still predominantly employ a valence-based framework, which classifies emotions by their general positivity or negativity to inform strategy generation. A key limitation of this kind of approach is its failure to account for the fact that the impact of emotions on decision-making behavior is highly contingent on the individual’s cognitive appraisal of the situation. As outlined in the Appraisal Tendency Framework, distinct emotions arise from different cognitive appraisal processes, and these processes shape how emotions influence judgments and behavioral choices in specific contexts. Consequently, further disaggregating emotions into specific cognitive dimensions can construct more adaptive models of emotional persuasion, offering greater explanatory power than traditional valence-based methods.

In light of the above analysis, this paper tries to address the limitations of traditional emotion modeling methods, which typically rely on a unidimensional view of emotion valence, by integrating the Appraisal Tendency Framework. Specifically, this study aims to decompose immediate emotions into their key cognitive dimensions and establish a mapping between emotional states and persuasion strategies based on these dimensions. The goal is to facilitate more rational strategy selection and behavioral responses in emotion-driven negotiation contexts.

### 2.4 The ATF in emotional persuasion

The Appraisal Tendency Framework (ATF), proposed by Lerner and Keltner [24], serves as a foundation for differentiating the effects of specific emotions on judgment and decision-making processes. According to the framework, distinct emotions trigger unique motivational processes, thereby elucidating the impact of each emotion type on decision-making outcomes. In the context of emotional persuasion, this framework suggests that an individual’s emotional response to a proposal influences their decision-making behavior (i.e., emotional persuasion strategy) through distinct cognitive appraisal tendencies, such as perceptions of certainty and pleasantness. Among the various prominent theories of cognitive appraisal, the theory proposed by Smith and Ellsworth [27] is particularly useful for our current concerns. According to the classic experiments of Smith and Ellsworth, all emotions could be described using a basic framework consisting of six cognitive dimensions: certainty, pleasantness, attentional activity, control, anticipated effort, and responsibility. These dimensions help to define and distinguish each discrete emotion, as well as to shape its possible influence on judgment and decision-making based on the ATF. However, the selection of cognitive dimensions when applying the Appraisal Tendency Framework largely depends on the specific research objectives and the context of practical application.

Research on emotional persuasion highlights the pivotal role of emotions and their impact in automated negotiation interactions [9,20,41]. In multi-round negotiations, emotional persuasion strategies often depend on the immediacy and intensity of emotional responses, while the certainty of the negotiation environment plays a crucial role in decision-making, particularly under time pressure. Importantly, it should be emphasized that in the context of business negotiation, emotions do not directly determine the choice of persuasion strategies. Instead, consistent with the core proposition of the ATF, emotions influence persuasion behavior by shaping the agent’s cognitive appraisal tendencies. That is, specific emotions trigger corresponding shifts in how individuals perceive certainty, pleasantness, control, and responsibility, which in turn affect their behavioral tendencies.

For instance, anger is typically viewed as a highly negative and high-certainty emotion, often linked to external attributions of blame [43]. The appraisals associated with anger—such as perceived certainty or control —promote confrontational tendencies, leading negotiators to adopt threat-based strategies. In such cases, the objective extends beyond reaching an agreement to include “suppressing” or dominating the opposing party through emotionally driven actions. Conversely, joy is generally associated with high pleasantness and certainty. When individuals experience joy, they tend to exhibit greater awareness of the current situation and a higher level of confidence in the negotiation process, which fosters cooperation and a win-win mindset [7]. These differentiated behavioral responses stem not directly from the emotions themselves, but from the distinct cognitive appraisals that each emotion activates.

In summary, this paper proposes combining the characteristics of emotional persuasion research with the understanding that emotions can be deconstructed into cognitive dimensions. By activating specific evaluative dimensions, such as pleasantness and certainty, corresponding decision-making modes can be triggered, thereby constructing a mapping from cognition to emotional persuasion strategy selection based on the Appraisal Tendency Framework.

### 2.5 Time in dynamic persuasion

Time is an essential element of negotiation. Most major reviews of the negotiation literature include time as a key variable [10]. There is evidence that time pressure can exacerbate any existing motivations in negotiations. That is, time pressure may be a double-edged sword, sometimes promoting cooperation and sometimes exacerbating hostility [44]. Pressure from time can have a universal influence on negotiation, for example, affecting the choice of negotiation strategies as well as basic psychological processes such as cognition and emotion [45].

In addition to the pressure from the respective deadlines of the negotiating parties, their attitudes toward time can affect the negotiation process in different ways. Firstly, the negotiators’ emotions constantly change according to the update of the other party’s proposal, and the update of the proposal takes time, so it is self-evident that the time factor affects the emotional persuasion process [29,30]. Secondly, negotiators have an objective cognitive bias towards time [46]. For instance, some negotiators may regard personal deadlines as a disadvantage, while others do the opposite, so that people with different cognitive tendencies may also adopt different time strategies in the same negotiation environment. In view of the above, both this disposition towards time and the actual deadline itself strongly influences the negotiation outcome. However, most existing models of an agent’s temporal attitudes are linear functions, and the gradient of the linear function is constant, which makes the agent’s concession strategy vulnerable to the adversary’s perception. Therefore, in this work, we construct a time function with a varying rate of change to describe the time pressure perceived by negotiators as the negotiation stage changes, so as to avoid their time-based concession behavior being detected.

## 3. The description of the proposed model

This paper addresses the problem of bilateral negotiations in the procurement environment, where both parties can negotiate over a range of product attributes. Each negotiating product attribute belongs to either a benefit type or a cost type, and the product attribute’s type depends on the position of an agent. The negotiation scenario can be a machine-to-machine interaction or a machine-to-human negotiation. In this paper, we concentrate on how to introduce human emotions and time beliefs into agent-based persuasive automated negotiation, i.e., we attempt to design a concession updating algorithm that considers agents’ emotion and time-related persuasion behavior with the goal of achieving better negotiation outcomes, including improved efficiency, fairness, and social welfare.

### 3.1 Model framework

In this study, automated negotiation is modeled as a complete negotiation episode, comprising multiple consecutive negotiation rounds. Each round is considered an independent “sub-phase,” during which the agent generates emotions, evaluates the situation cognitively, and makes strategic decisions based on real-time perceptions of the environment. To address this, we propose a state-dependent concession updating algorithm designed to dynamically adjust the agent’s proposal behavior during each negotiation round. The algorithm operates on a round-by-round basis, with time as the unit of measurement, and integrates two critical state variables: the agent’s emotional state and its temporal attitude.

First, when an agent receives a proposal from the opponent in a negotiation round, it generates an emotional response based on its internal emotion-generation model. This emotion arises from the deviation between the agent’s subjective assessment of the opponent’s current proposal and its expected value. This moment is the specific time emphasized by the ATF theory—namely, the critical cognitive window in which emotions influence judgment and decision-making. The model focuses on the immediate emotional impact on decision-making during each round, rather than modeling the cumulative evolution of emotions throughout the entire negotiation process.

Second, the role of time in negotiation behavior is critical. An agent’s subjective attitude toward time (e.g., urgency or patience) can influence the magnitude of its eventual concessions by affecting expectations, risk assessments, and motivational dispositions. Previous studies have also demonstrated that time beliefs in negotiation activate various psychological and motivational mechanisms [10], which significantly influence negotiation behavior [12]. As such, time attitudes are incorporated into our model’s state-dependent update function as a key variable affecting the adjustment of emotional persuasion and concession strategies.

In the modeling process, emotional factors and temporal factors are considered independently. This design is consistent with cognitive load theory [47]: when handling complex tasks, humans tend to process different types of information through separate cognitive systems. In negotiation contexts, emotional evaluation primarily reflects the agent’s immediate reaction to the opponent’s proposal, while temporal evaluation captures the agent’s subjective perception of the negotiation pace and its concession adjustments. Based on these differentiated characteristics, this study adopts a relatively independent modeling approach for the two factors. This not only ensures computational efficiency but also enhances theoretical interpretability.

**Fig 1:**
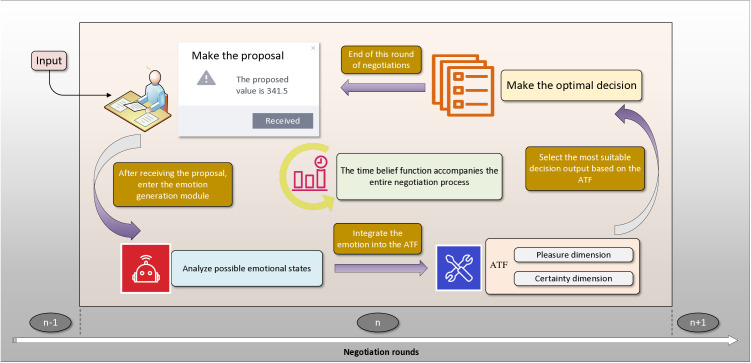
The concessions dependent on the emotion and time beliefs framework.

As such, a concession in this paper is believed to have three components: the effect of the agent’s emotional state, the effect of the agent’s time perception, and the basic concession magnitude. The former two effects are modeled to adjust the concession magnitude to arrive at a new concession step each round. To more clearly illustrate how these mechanisms impact agent decisions in multiple rounds of emotion-driven negotiation, we present the concessions dependent on the emotion and time beliefs framework (see Fig 1). This framework explicitly demonstrates how the agent dynamically adjusts the concession magnitude and generates the updated proposal in each round by incorporating immediate emotional responses and perceptions of time.

To validate the proposed model, this paper adopts a comprehensive research approach that integrates both qualitative and quantitative methods. The qualitative component employs agent-based modeling and logical deduction to examine the underlying mechanisms of concession behavior in automated negotiation. Specifically, it investigates how key cognitive dimensions influence the selection of emotional persuasion strategies within the Appraisal Tendency Framework. The quantitative component systematically evaluates and compares the model’s performance across various multi-dimensional indices, including negotiation success rate, joint utility, utility disparity, and negotiation efficiency. This evaluation is conducted through simulation, statistical analysis, and case studies, aiming to quantitatively assess the model’s validity, applicability, and its comparative advantage in real-world negotiation contexts.

In addition, the key variables and notations involved in the modeling process are summarized in Table 1. It should be noted that the methods and experiments in this paper do not involve human participants or animals.

**Table 1:** Definition and description of key variables and notations.

	Notation	Definition and Description
Agent features	agent i	i = “buyer” for buyer agent and i = “seller” for seller agent
	$C_{i}$	agent i ’s trustworthiness, i.e., the quality of being reliable and honest
Round features	$U_{i}^{t}$	agent i ’s real utility, i.e., the utility value calculated from the opponent’s real offers
	$U_{i}^{\prime t}$	agent i ’s expected utility, i.e., the utility value calculated based on one’s own expectations of the final deal
	$P_{i,k}\left(n\right)$	agent i ’s offer for attribute k in the n round
	$P(n{)}_{k}^{*}$	the attribute value when the persuasion is successful in the n round
Emotional states	$E_{i}^{t}$	agent i ’s intensity of pleasantness, i.e., the strength of an agent’s pleasantness
Attribute features	${\bar{x}}_{k}$	the maximum value $x_{k}$ on attribute k
	${\underset{―}{x}}_{k}$	the minimum value $x_{k}$ on attribute k
	$P_{i,k}(n{)}^{\max}$	the maximum value of the range acceptable to agent i in the n round
	$P_{i,k}(n{)}^{\min}$	the maximum value of the range acceptable to agent i in the n round
	$Q_{i,k}\left(n\right)$	agent i ’s expected added value from attribute k if the negotiation is successful in the n round, i.e., the increment of each new proposal relative to the boundary value of the proposal
Time-related states	$T_{i}$	agent i ’s maximum number of negotiation rounds
	$f_{i}(n)$	agent i ’s time function, i.e., the agent’s attitude toward negotiation time

### 3.2 Emotional persuasion strategy selection modeling

Based on the theory of the Appraisal Tendency Framework, we establish an emotional persuasion concession strategy selection model that considers an agent’s two core cognitive dimensions, namely, certainty and pleasantness. Specifically, this section first describes the utility function applied in this paper, and then, generate an emotional response within the negotiation process. Next, it models and quantifies the agent’s dimensions of certainty and pleasantness. Finally, the agent’s current emotion is assessed cognitively using the ATF, which informs the selection and adjustment of emotional persuasion strategies based on these core appraisal dimensions.

#### 3.2.1 Agent’s utility function.

The utility is regarded as the satisfaction level that the focal agent gets from the opponent’s offer in the current round. In this paper, we focus on a bilateral multi-attribute negotiation with different attribute types. In a multi-attribute negotiation, an agent’s total utility is the weighted sum of the utilities of each individual attribute and the agent would like to make the total utility as large as possible. However, due to the agent’s position in negotiation, e.g., the buyer or the seller, the utility of an individual attribute may be positively or negatively related to the attribute value. As such, each attribute can be classified as either a benefit type or a cost type [48,49]. An agent will get more utility from a larger value of a benefit-type attribute. By contrast, an agent will get more utility from a smaller value of a cost-type attribute. For example, a buyer agent views the price as a cost-type attribute, whereas a seller agent views it as a benefit-type attribute.

Assume that a buyer agent and a seller agent are involved in a negotiation. The focal negotiation agent’s utility considers its initial and reserved offers for the negotiation attributes, as well as the opponent’s offers for the current round. When the focal agent receives a proposal including the opponent agent’s proposed value for each negotiating attribute, the focal agent i (i = “buyer” or “seller”) will get a utility of $U_{i}^{t}$.

Combined with the classification of negotiation attributes, the agent i ’s utility $U_{i}^{t}$ can be calculated in terms of (1)and(2):


$$U_{i}^{t}=\sum {\mathrm{\backslash nolimits}}_{k=1}^{n}w_{ik}u_{ik}$$ (1)

$$u_{ik}=\left\{ \begin{array}{cc} \frac{x_{k}-{\underset{―}{x}}_{k}}{{\bar{x}}_{k}-{\underset{―}{x}}_{k}}, & \text{benefit-type}\\ \frac{{\bar{x}}_{k}-x_{k}}{{\bar{x}}_{k}-{\underset{―}{x}}_{k}}, & \text{cost-type} \end{array}\right.\;$$ (2)

where $w_{ik}$ represents the weight that agent i assigns to attribute k (k=1,2,⋯,n), and $u_{ik}$ represents agent i ’s utility of the opponent agent’s proposed value $x_{k}$ on attribute k. Besides, ${\bar{x}}_{k}$ and ${\underset{―}{x}}_{k}$ are the attribute’s corresponding maximum and minimum values.

#### 3.2.2 Agent’s certainty dimension.

In agent-based automated negotiation, an agent will inevitably face varying degrees of uncertainty generated by the opponent’s behavior, and in order to reduce this uncertainty, i.e., enhance certainty, it is necessary to select the most trustworthy agent from a large pool of potential candidate agents. Therefore, an agent’s certainty dimension in this paper is measured by the level of its trust. Trust is the premise of an agent’s persuasion and measures the degree to which the agent can be relied on to conduct transactions [50]. Ramchurn et al. [17] endowed agents with decision making models that exploit the notion of trust and persuasive techniques during the negotiation process to reduce the level of uncertainty and achieve better deals in the long run. Generally speaking, the certainty of an agent is largely constrained by the limited cognition and future direction of action, whereas trust can motivate an agent to obtain the bond of connection and stable support in the uncertain environment, thus enhancing certainty [51]. For example, if an agent holds a high level of trust in the opponent agent, it will believe that the opponent agent will behave consistently and can handle contingencies well during the negotiation, and accordingly the agent will perceive a high level of certainty in the negotiation.

This paper adopts the rating method to model an agent’s trust, i.e., the certainty dimension. Rating is the most commonly used trust level modeling method in intelligent negotiation [52,53]. In specific, we denote an agent’s trustworthiness as $C_{i}\in [0,1]$. Divide $C_{i}$ into 3 levels and the cut-off points are denoted as $c_{1}$, $c_{2}$ in an increasing order. They correspond to fuzzy intervals of high, middle, and low trust levels, which can best characterize the agent’s trust level in uncertain environments. The values of $c_{1}$ and $c_{2}$ can be determined with reference to [16], which employs the Naive Bayes algorithm to classify trust relationships into high, medium, and low levels, yielding a generalized classification standard. Table 2 displays the division of the ratings.

**Table 2:** Modeling the certainty level.

Level of certainty	Interval of trustworthiness
High	$\left(c_{\text{2}},\text{1}\right]$
Mid	$\left(c_{\text{1}},c_{\text{2}}\right]$
Low	$\left[\text{0},c_{\text{1}}\right]$

#### 3.2.3 Agent’s pleasantness dimension.

As another kind of agent’s core appraisal dimension for emotion generation, pleasantness can be identified as a feeling caused by external stimuli [54]. Assume that a buyer agent and a seller agent are involved in a negotiation. When receiving a proposal that includes the opponent agent’s proposed value for each negotiating attribute, the focal agent i (i = “buyer” or “seller”) will get a real utility of $U_{i}^{t}$.Meanwhile, agent i can also get an expected utility of $U_{i}^{\prime t}$ because it has an expected deal value for each negotiating attribute. The deviation of $U_{i}^{t}$ from $U_{i}^{\prime t}$ can be viewed as the stimuli which leads to agent i ’s generation of pleasantness. The positive deviation implies that the proposal exceeds agent i ’s expectation and positive pleasantness will consequently be generated. Conversely, the negative deviation implies that the proposal cannot meet the agent’s expectation and accordingly negative pleasantness will be generated.

We use the intensity of pleasantness to characterize the strength of an agent’s pleasantness. Here, the Weber-Fechner Law is applied to depict an agent’s intensity of pleasantness by the following(3):


$$E_{i}^{t}=\tanh\left(k_{m}\ln\left(1+\frac{U_{i}-{U^{\prime }}_{i}}{{U^{\prime }}_{i}}\right)\right)$$ (3)

where $k_{m}$ is a scale coefficient, and the function tanh(·) is used to normalize the value of $E_{i}^{t}$ to be in (−1,1).

The Weber-Fechner Law originally describes a relationship between a human’s perception of a stimulus and the stimulus’ physical strength [55]. Under this law, a human’s perception of a stimulus varies with the logarithm of the ratio of the physical strength of the stimulus to a threshold that the stimulus has to overcome to be perceived. An agent’s pleasantness resulting from the comparison between its utility of the opponent agent’s proposal (i.e., the actual utility) and the focal agent’s expected utility follows this law. The more the actual utility deviates from the expected one, the more strength of positive or negative pleasantness an agent will perceive. When the actual utility firstly surpasses or falls behind the expected one, an agent will be most sensitive to the difference and thus perceive the largest change in the strength of pleasantness. As the difference enlarges, an agent will be insensitive to the difference and as a result the perceived change in the strength of pleasantness will tail off.

#### 3.2.4 Agent’s emotion generation.

In this paper, we adopt the emotion generation method based on the core affect theory proposed by Wu et al. [16] as the intrinsic emotion generation model for the negotiation agent. This model is used to generate the agent’s real-time emotion during each negotiation round. According to Wu et al. [16], the intrinsic emotion generation model quantifies the emotions of the agent along two dimensions: the value dimension and the arousal dimension. Specifically, these dimensions are assessed through the agent’s subjective evaluation of two factors: the direct stimulus from the opponent’s proposal and the activation level of the agent itself. This model enables the emotional modeling of the agent’s current state, thereby providing the agent with a distinctive emotional experience.

According to Wu et al.‘s study, once the agent is activated, it enters an aroused state in the context of automated negotiation. Therefore, the emotion of the negotiating agent is primarily influenced by its subjective evaluation of the opponent’s offer. This evaluation, denoted as $O_{i}(t)$, is derived by comparing agent i’s expected offer to the actual offer received from opponent j. The corresponding formula for this calculation is as follows:


$$O_{i}(t)=\{\begin{array}{cc} {}*20c\frac{{\overset{\wedge }{P}}_{i}(t)-P_{j}(t)}{{\overset{\wedge }{P}}_{i}(t)}, & cost-type\\ -\frac{{\overset{\wedge }{P}}_{i}(t)-P_{j}(t)}{{\overset{\wedge }{P}}_{i}(t)}, & benefit-type \end{array}$$ (4)

where $\overset{\wedge }{\underset{i}{P}}(t)$ represents Agent i’s expected offer in round t, and $P_{j}(t)$ denotes the actual offer made by opponent j in round t. Based on this, the basic emotion generation function $e_{i}(t)$ of the agent in round t, as proposed by Wu et al. [16], can be derived and is calculated as follows:


$$e_{i}(t)=O_{i}(t)\times \sigma_{i}$$ (5)

Herein, $O_{i}(t)$ represents the subjective evaluation generated by the agent based on the negotiation interaction stimuli, and $\sigma_{i}$ denotes the activation level. While emotions are inherently continuous, to facilitate computational modeling in behavior, Wu et al. further mapped the agent’s emotional state to the six basic emotional expressions identified by Ekman [56]. These six basic emotions, spanning from negative to positive, are primarily employed to enhance emotional expressiveness and anthropomorphism during agent interactions.

However, at the level of strategic behavior, some of the basic emotions identified by Ekman et al. do not, on their own, provide significant guidance for adjustments to specific emotional persuasion strategies. Consequently, building on the empirical analysis of emotions in negotiation contexts from existing studies, we adapted the original emotion system to include a set of emotions that are more immediate and strategy-directed in the context of emotional persuasion research. These emotions—anger, disappointment, anxiety, anticipation, gratification, and joy—are characterized by stronger immediate reactivity and greater differentiation in cognitive evaluation dimensions, making them more suitable for analyzing the impact of emotions on agents’ persuasive strategy choices.

#### 3.2.5 Emotional persuasion strategy selection based on the ATF.

In the negotiation process, emotional persuasion is widely acknowledged as a crucial strategy that can significantly influence the behavior of the opposing party [43,57]. One party may initiate emotional persuasion by leveraging emotional expressions to encourage the other party to make concessions in its favor. Conversely, the opposing party may respond with counter-proposals through responsive emotional expression. In this interactive process, we introduce the Appraisal Tendency Framework, which focuses on analyzing immediate emotional responses during each round of negotiation and further decomposes these emotions into their core cognitive appraisal dimensions, providing a foundation for the dynamic adjustment of emotional persuasion strategies [58].

As outlined in Section 3.2.4, during the negotiation process, the agent’s internal emotion model generates real-time emotional responses based on the discrepancy between the opponent’s proposal and the agent’s expectations. These immediate emotions include anger, disappointment, anxiety, anticipation, gratification, and joy. These emotional reactions not only reflect the agent’s response to the negotiation dynamics but also serve as critical factors that influence strategy selection and concession behavior. According to the ATF, once emotions are activated, a series of cognitive tendencies are triggered, prompting the agent to evaluate the current and future negotiation scenarios based on the appraisal dimensions involved, thereby shaping their strategic decision-making behavior.

On this basis, this paper maps the six emotions outlined above to the two key cognitive dimensions of the ATF: pleasantness and certainty. It then analyzes these emotions within a two-dimensional cognitive space. For example, anger typically represents an emotion with highly negative pleasantness and high certainty, often accompanied by strong aggressive tendencies. In contrast, anxiety, characterized by lower negative pleasantness and low certainty, tends to involve internal attributions and is typically non-aggressive. Furthermore, although both joy and gratification are positive emotions, joy is associated with a higher degree of certainty, making it more likely to promote cooperative and trusting behaviors. Variations in the distribution of these emotions across the pleasantness and certainty dimensions help predict differing behavioral tendencies in negotiation decisions.

To enhance the mapping between emotions and behavioral strategies, we extend the basic persuasion strategies used in the existing literature [20,59] to a total of six types: reward type, appeal type, analog type, explanatory type, complaint type, and threat type. The definition of each type is given as follows:

- The *threat-type* persuasion is defined as that an agent forces the opponent agent to make concessions via valid threats.
- The *complaint-type* persuasion refers to that an agent persuades the other party by its own difficulties and sufferings.
- The *explanatory-type* persuasion is defined as that an agent explains its proposal and expounds its reasons for the proposal.
- The *appeal-type* persuasion means that an agent persuades the opponent agent by calling for both parties to follow historical behavior or current popular practices.
- The *analog-type* persuasion refers to that an agent shows the merits of its proposal by comparing its proposal with the opponent’s.
- The *reward-type* persuasion refers to that an agent encourages the opponent agent to make concessions by promising to give the latter a certain reward.

To theoretically justify the mapping between specific emotions and corresponding persuasion strategies, we draw on findings from psychology and communication research. Anger is considered a highly negative and high-certainty emotion, typically linked to external attribution of blame. It tends to trigger approach-oriented and confrontational behaviors, making it particularly aligned with threat-based persuasion strategies [60,61]. Disappointment is associated with increased sensitivity to loss, a desire for compensation, and the expression of dissatisfaction, which corresponds well to complaint-type strategies that emphasize personal hardship and grievance [62]. Anxiety, rooted in low certainty and internal attribution, induces risk aversion and a strong desire for clarity [63]. Therefore, explanatory strategies that provide justification and reduce ambiguity are most suitable in anxious states. Anticipation, which reflects a forward-looking focus on potential rewards under uncertain conditions [64], is best supported by appeal-type strategies that invoke historical cooperation and social norms to foster trust and stability [65]. Gratification, as a moderately positive and cooperative emotion, encourages consensus-seeking behavior and fits well with analogical strategies that emphasize comparative advantage and rational evaluation [66]. Lastly, joy, often characterized by high pleasantness and high certainty, enhances openness and willingness to compromise, thereby aligning naturally with reward-based persuasion strategies aimed at building mutual trust and benefit [43].

Building on the analysis above and combined with the ATF theory, the paper constructs a three-layer mapping mechanism, progressing from emotions to cognitive dimensions, and subsequently to strategy matching. The proposed model essentially represents a continuous emotion-to-continuous strategy mapping framework, where the intensity of emotional states can lead to nuanced strategy adaptations. For example, anger may range from mild to intense, potentially triggering a spectrum of threat-based strategies from subtle warnings to overt confrontation. However, to enhance interpretability and operational clarity, this study adopts a discrete approximation of this mechanism—mapping identifiable emotional states to representative persuasive strategies. Table 3 summarizes this mapping, outlining the characteristics of six emotions in the cognitive dimension space, their associated decision-making tendencies, and the corresponding emotional persuasion strategies.

**Table 3:** Emotion, pleasantness, certainty, decision impact, and potential strategies.

Emotion	Pleasantness	Certainty	Decision Impact	Potential Strategy
Anger	Negative, High	High	Increased risk preference, external attribution	Threat-type
Disappointment	Negative, Med	Med	Increased loss sensitivity, seeks compensation	Complaint-type
Anxiety	Negative, Low	Low	Risk-averse tendency, seeks security	Explanatory-type
Anticipation	Positive, Low	Low	Future gain orientation, tends toward compromise	Appeal-type
Gratification	Positive, Med	Med	Increased willingness to cooperate, accepts moderate concessions	Analog-type
Joy	Positive, High	High	Overly optimistic, may compromise too quickly	Reward-type

In summary, based on the ATF, when the agent perceives the current proposal positively and the negotiation environment exhibits a certain degree of certainty, the agent is more likely to adopt a positive and friendly emotional persuasion strategy. In contrast, when the agent’s value perception is negative and the environment is characterized by greater certainty, the agent is more inclined to employ aggressive, pressure-driven strategies, with the aim of enhancing negotiation control or exerting greater influence on the counterpart. Generally, the use of a positive emotional strategy indicates the agent’s greater willingness to cooperate and a propensity to make more concessions. In contrast, the adoption of highly negative emotional strategies signals a lack of genuine willingness to cooperate and a tendency to make fewer concessions [43].

### 3.3 The composition of emotional persuasion

#### 3.3.1 Basic concession magnitude.

Consider the case where a buyer agent and a seller agent are engaged in negotiation about attribute k (k=1,2,⋯,K). An agent’s offer cannot be accepted by the opponent agent unless the offered value is within the latter agent’s acceptable ranges. Because of the concessions made by both agents, the acceptable ranges are changed round by round. Assume $P_{i,k}(n{)}^{\max}$ and $P_{i,k}(n{)}^{\min}$ to be the maximum value and the minimum value of the range acceptable to agent i(i=sorb) in the n−th round, where s and b stand for the seller and the buyer, respectively. A necessary condition for a negotiation to reach consensus is that $P_{i,k}(n{)}^{\max}$ and $P_{i,k}(n{)}^{\min}$ are non-increasing and non-decreasing in n, respectively. In fact, the two boundaries can take the values of the seller agent’s and the buyer agent’s proposals. For example, for a benefit-type attribute, $P_{s,k}(n{)}^{\max}$ is $x_{s,k}\left(n\right)$, which is the seller agent’s proposal in the n−th round and $P_{s,k}^{\min}(n)$ is $x_{b,k}\left(n\right)$, which is the buyer agent’s proposal.

Here, an added value is defined as an increment for each new proposal compared to the boundary value of the proposal. For example, let $P(n+1{)}_{k}^{*}$ represent the attribute value when the persuasion is successful in the (n+1)−th round, and then, compared to the first proposal, an agent can get an added value $r_{i,k}\left(n+1\right)$ from attribute k by equation (6).


$$\begin{array}{c} r(n+1{)}_{i,k}=\{\begin{array}{cc} {}*20lP(n+1{)}_{k}^{*}-P(1{)}_{i,k}^{\min}, & benefit-type\\ P(1{)}_{i,k}^{\max}-P(n+1{)}_{k}^{*}, & cost-type \end{array}\\ \;i=s\;or\;b \end{array}$$ (6)

Given that the negotiation is successful, the worst value that an agent can obtain on attribute k is $P_{i,k}(1{)}^{\min}$ if this attribute is a cost-type. Since the consensus value is not larger than the worst one, the difference can be viewed as the agent’s reward. Similarly, if attribute k is a benefit-type, the worst value that the agent can obtain is $P_{i,k}(1{)}^{\max}$. Since the consensus value is not smaller than the worst one, the agent can obtain this difference as the added value. At the end of the n−th round, an agent has a belief of the consensus value of attribute k which belongs to $\left[P_{i,k}(n{)}^{\min},P_{i,k}(n{)}^{\max}\right]$ for the next round. Then, the agent’s expected added value $Q_{i,k}\left(n+1\right)$ if the negotiation is successful in the (n+1)−th round is formulated as(7).


$$\begin{array}{c} Q_{i,k}(n+1)=\{\begin{array}{cc} {}*20l\int_{P_{i,k}{\left(n\right)}^{\min}}^{P_{i,k}{\left(n\right)}^{\max}}\left(x-P_{i,k}{\left(1\right)}^{\min}\right)p_{i,k}(x)dx, & benefit-type\\ \int_{P_{i,k}{\left(n\right)}^{\min}}^{P_{i,k}{\left(n\right)}^{\max}}\left(P_{i,k}{\left(1\right)}^{\max}-x\right)p_{i,k}(x)dx, & cost-type \end{array}\\ \;i=s\;or\;b,n=1,2,\cdots ,N \end{array}$$ (7)

where $p_{i,k}(x)$ is the density function of the agent’s belief of the consensus value of attribute k. The agent takes this expected reward as the basic concession magnitude for the next-round offer. As such, the basic concession magnitude can be dynamically changed as persuasion proceeds.

#### 3.3.2 Emotion-related concession behavior.

When emotional persuasion is triggered, the buyer (seller) agent will also perceive the other party’s emotional persuasion when receiving the other party’s proposal. Therefore, the buyer (seller) agent’s concessions will be affected by the other party’s emotional persuasion. The degree of this influence denoted as α is called the emotional persuasion factor.

Here we give an example to illustrate this process. According to the selection rules of emotional persuasion strategies in Table 3, an agent can generate corresponding emotional persuasion strategies based on their two cognitive dimensions (i.e., certainty and pleasantness). But depending on the role (buyer or seller agent), an agent has its own sensitivity to the opponent’s expression of emotions, i.e., the emotional persuasion factor α. Before the negotiation starts, the certainty dimension can be obtained by trust rating, i.e., the certainty level of both parties is public information. When the negotiation is in progress, the agent can calculate the opponent’s pleasantness dimension by taking the opponent’s first round offer as the opponent’s expected value and combining it with its latest offer. Then, the agent will complete the detection of the opponent’s emotional strategy based on its own cognitive system (the Appraisal Tendency Framework). Finally, the focal agent makes a concession based on the detection results. For example, when the focal agent senses a possible threat strategy from the adversary, indicating that the opponent’s sincerity of cooperation is already low, the focal agent may continue to reduce its concessions; but if a possible complaint strategy is detected from the adversary, the focal agent may make more concessions to pacify the opponent (i.e., to avoid making the opponent more frustrated).

#### 3.3.3 Timing-related concession behavior.

This paper constructs a time function with a non-constant rate of change by analyzing the negotiator’s time-based concession attitudes. An agent’s time beliefs have previously been mainly modeled in three forms [29,30,67]: a fixed constant, a linear increase, and a linear decrease in the rounds of negotiation. The first form indicates that the degree of compromise in an agent’s concession is a constant. The second form indicates that the degree of compromise in an agent’s concession gradually increases, and the third form indicates that the magnitude of an agent’s concession gradually decreases. It is obvious that the latter two forms of time beliefs are an improvement over the first form. Nevertheless, the linear time function is easily detectable by the adversary during the concession process. The time function with a changing rate proposed by this paper can reduce this limitation to a certain extent. Meanwhile, this proposed time function can capture how a negotiator’s perceived time pressure varies with the stages of the negotiation in a real-world scenario.

**Fig 2:**
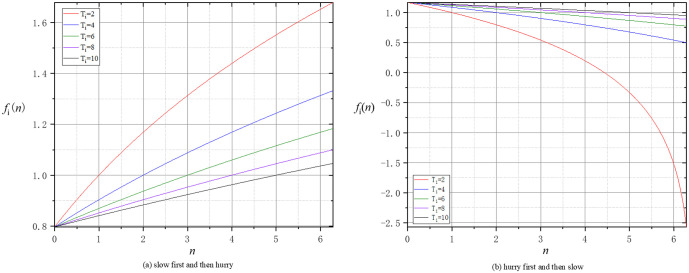
The agent’s concession tendency under the time function.

In making concessions, an agent may adopt a tentative attitude in the initial rounds of negotiation and accordingly may choose a relatively small concession; after a while, in order to reach an agreement as soon as possible, the agent may select a large concession. On the contrary, an agent may select a large concession in the initial rounds; as the persuasion proceeds and the negotiation approaches an agreement, the agent may reduce the concession. Based on the above analysis and existing studies [29,30], we consider the dynamic change of an agent’s attitude toward time pressure at different stages of negotiation and thus construct the time function $f_{i}(n)$ to characterize the above two timing-related persuasion behavior.


$$\begin{array}{c} f_{i}(n)=\{\begin{array}{cc} {}*20l\beta_{i}\times \ln(e+\frac{n}{T_{i}}-\Gamma_{i}), & slow\;first\;and\;then\;hurry\\ \beta_{i}\times \ln(e+\Gamma_{i}-\frac{n}{T_{i}}), & hurry\;first\;and\;then\;slow \end{array}\\ \;i=s\;or\;b \end{array}$$ (8)

where e is the natural constant, $T_{i}$ represents the maximum number of rounds of negotiation set by the buyer agent or seller agent, and $\beta_{i}$ and $\Gamma_{i}$ are both individual parameters representing the actual preferences of various agents. The flexibility in adjusting parameter $\beta_{i}$ empowers agents to customize the rate of change of the time function according to their personal preferences. The parameter $\Gamma_{i}$ can also be flexibly adjusted to allow the agent to adjust the initial concession benchmark according to individual preferences. Assuming $\beta_{i}$ = 1 and $\Gamma_{i}$ = 0.5, Fig 2 illustrates the tendency of concessions over time for agents with different time attitudes under different negotiation time limits.

#### 3.3.4 Concession process pseudocode.

To present the concession process more clearly, the corresponding pseudocode is provided in Table 4.

**Table 4:** Concession Updating Procedure.

Algorithm: Concession Updating Procedure (Emotion–Time Driven)
**Input:**
Previous offer $P_{i,k}(n)$
previous acceptable interval boundaries $P_{i,k}(n{)}^{\min}$ and $P_{i,k}(n{)}^{\max}$
Trust level $C_{i}$
Expected utility $U_{i}^{\prime t}$
Real utility $U_{i}^{t}$
Maximum negotiation rounds $T_{i}$
**Output:**
Current offer $P_{i,k}\left(n+1\right)$; if the stopping condition is met, output agreement values $P(n{)}_{k}^{*}$
**Process:**
For n = 1, 2, …, $T_{i}$ do Step 1: Compute basic concession For each attribute k do Based on the last acceptable interval $P_{i,k}(n{)}^{\min}$ and $P_{i,k}(n{)}^{\max}$ compute $Q_{i,k}\left(n\right)$ End for Step 2: Emotion adjustment via ATF mapping (Eq. (3) + Table 3) Compute pleasantness intensity $E_{i}^{t}$ Step 3: Time adjustment function Compute time function $f_{i}(n)$ Step 4: Update offers For each attribute k do If attribute k is cost-type then Update $P_{i,k}(n)$ using Eq. (10) or Eq. (11) according to time attitude Else// attribute k is benefit-type Update P_{i,k}(n) using Eq. (10) or Eq. (11) according to time attitude End if End for Step 6: Termination check and agreement If for all k, |$P_{s,k}(l)$ - $P_{b,k}(l)$ | < 1 then $P_{k}^{*}$ ← ($P_{s,k}(l)$ +$P_{b,k}(l)$)/ 2 Output $P_{k}^{*}$ and stop End if

## 4. Model analysis

Given the above modeling, we can now construct a function for an agent to update the proposal. This offer updating function consists of two parts, namely the proposed value in the previous round and the concessions generated for the current round. Assume that agent i (i=s or b) made an offer $P_{i,k}\left(n-1\right)$ for attribute k in the previous round, and the agent’s offer for this attribute in the n−th round is calculated by equation (9).


$$\begin{array}{c} P_{i,k}(n)=\{\begin{array}{cc} {}*20lP_{i,k}(n-1)+\alpha_{i}(n)f_{i}(n)Q_{i,k}(n), & cost-type\\ P_{i,k}(n-1)-\alpha_{i}(n)f_{i}(n)Q_{i,k}(n), & benefit-type \end{array}\\ \;i=s\;or\;b \end{array}$$ (9)

The corresponding expressions for $f_{i}(n)$ can be obtained from Equation(8). As for $Q_{i,k}(n)$, in the case of incomplete information, negotiating agents generally believe that each value in their last round of proposal intervals has an equal chance of being chosen as the current agreed-upon attribute value. Thus, the choice of the agreed value follows a uniform distribution with a density of $p_{i,k}(x)=\frac{\text{1}}{P_{i,k}{\left(n-1\right)}^{\max}-P_{i,k}{\left(n-1\right)}^{\min}}$ for the agent. Then, according to the corresponding expressions for $Q_{i,k}(n)$ from Equation (7), we have:


$$\begin{array}{cc} & Q_{i,k}(n)\text{=}\int_{P_{i,k}{\left(n-1\right)}^{\min}}^{P_{i,k}{\left(n-1\right)}^{\max}}\left(x-P_{i,k}{\left(1\right)}^{\min}\right)\frac{1}{P_{i,k}{\left(n-1\right)}^{\max}-P_{i,k}{\left(n-1\right)}^{\min}}dx\\ & =\frac{\frac{1}{2}x^{2}-P_{i,k}{\left(1\right)}^{\min}x}{P_{i,k}{\left(n-1\right)}^{\max}-P_{i,k}{\left(n-1\right)}^{\min}}|P_{i,k}{\left(n-1\right)}^{\min}P_{i,k}{\left(n-1\right)}^{\max}\\ & =\frac{\frac{1}{2}{\left(P_{i,k}{\left(n-1\right)}^{\max}\right)}^{2}-P_{i,k}{\left(1\right)}^{\min}\times P_{i,k}{\left(n-1\right)}^{\max}}{P_{i,k}{\left(n-1\right)}^{\max}-P_{i,k}{\left(n-1\right)}^{\min}}-\frac{\frac{1}{2}{\left(P_{i,k}{\left(n-1\right)}^{\min}\right)}^{2}-P_{i,k}{\left(1\right)}^{\min}\times P_{i,k}{\left(n-1\right)}^{\min}}{P_{i,k}{\left(n-1\right)}^{\max}-P_{i,k}{\left(n-1\right)}^{\min}}\\ & =\frac{\frac{1}{2}\left[{\left(P_{i,k}{\left(n-1\right)}^{\max}\right)}^{2}-{\left(P_{i,k}{\left(n-1\right)}^{\min}\right)}^{2}\right]-P_{i,k}{\left(1\right)}^{\min}\times (P_{i,k}{\left(n-1\right)}^{\max}-P_{i,k}{\left(n-1\right)}^{\min})}{P_{i,k}{\left(n-1\right)}^{\max}-P_{i,k}{\left(n-1\right)}^{\min}}\\ & =\frac{\frac{1}{2}\left[(P_{i,k}{\left(n-1\right)}^{\max}+P_{i,k}{\left(n-1\right)}^{\min})\times (P_{i,k}{\left(n-1\right)}^{\max}-P_{i,k}{\left(n-1\right)}^{\min})\right]-P_{i,k}{\left(1\right)}^{\min}\times (P_{i,k}{\left(n-1\right)}^{\max}-P_{i,k}{\left(n-1\right)}^{\min})}{P_{i,k}{\left(n-1\right)}^{\max}-P_{i,k}{\left(n-1\right)}^{\min}}\\ & =\frac{\left(P_{i,k}{\left(n-1\right)}^{\max}-P_{i,k}{\left(n-1\right)}^{\min})\times (\frac{1}{2}(P_{i,k}{\left(n-1\right)}^{\max}+P_{i,k}{\left(n-1\right)}^{\min})-P_{i,k}{\left(1\right)}^{\min}\right)}{P_{i,k}{\left(n-1\right)}^{\max}-P_{i,k}{\left(n-1\right)}^{\min}}\\ & =\frac{1}{2}(P_{i,k}{\left(n-1\right)}^{\max}+P_{i,k}{\left(n-1\right)}^{\min})-P_{i,k}{\left(1\right)}^{\min}>0 \end{array}$$

On this basis, if the time belief is “slow first and then fast”, the function of offer updating can be further refined by equation (10); otherwise, it can be refined by equation (11):


$$\begin{array}{c} P_{i,k}(n)=\{\begin{array}{cc} {}*20lP_{i,k}(n-1)+\alpha_{i}(n)\ln(e+\frac{n}{T_{i}}-0.5)\left[\frac{\text{1}}{\text{2}}(P_{i,k}{\left(n-1\right)}^{\max}+P_{i,k}{\left(n-1\right)}^{\min})-P_{i,k}{\left(1\right)}^{\min}\right], & cost-type\\ P_{i,k}(n-1)-\alpha_{i}(n)\ln(e+\frac{n}{T_{i}}-0.5)\left[\frac{\text{1}}{\text{2}}(P_{i,k}{\left(n-1\right)}^{\max}+P_{i,k}{\left(n-1\right)}^{\min})-P_{i,k}{\left(1\right)}^{\min}\right], & benefit-type \end{array}\\ \;i=s\;or\;b \end{array}$$ (10)

$$\begin{array}{c} P_{i,k}(n)=\{\begin{array}{cc} {}*20lP_{i,k}(n-1)+\alpha_{i}(n)\ln(e+0.5-\frac{n}{T_{i}})\left[\frac{\text{1}}{\text{2}}(P_{i,k}{\left(n-1\right)}^{\max}+P_{i,k}{\left(n-1\right)}^{\min})-P_{i,k}{\left(1\right)}^{\min}\right], & cost-type\\ P_{i,k}(n-1)-\alpha_{i}(n)\ln(e+0.5-\frac{n}{T_{i}})\left[\frac{\text{1}}{\text{2}}(P_{i,k}{\left(n-1\right)}^{\max}+P_{i,k}{\left(n-1\right)}^{\min})-P_{i,k}{\left(1\right)}^{\min}\right], & benefit-type \end{array}\\ \;i=s\;or\;b \end{array}$$ (11)

To sum up, it is clear that a new proposal is influenced not only by the basic negotiation process factors such as the proposals of the two negotiating parties in the last round, i.e., $P_{i,k}(n-1{)}^{\max}$ and $P_{i,k}(n-1{)}^{\min}$, and the worst value of the proposal $P_{i,k}(1{)}^{\min}$ in the first round, but also by the environmental stimuli (the opponent’s emotions) and the time beliefs of the negotiating agent itself. Among them, with the progress of negotiation, $P_{i,k}(n-1{)}^{\max}$ and $P_{i,k}(n-1{)}^{\min}$ are constantly updated and derived from the maximum and minimum values in the proposal interval of both parties in the n−1 round of negotiation, respectively; the value of $\alpha_{i}(n)$ is influenced by the opponent’s emotion specifically based on the emotional persuasion strategy selection rule (refer to Table 3); and the negotiators’ attitude towards the time will also change with the progress of negotiation. This can indicate that the proposed model, on the basis of following the objective conditions of negotiation, also considers the environmental factors and the individual needs of negotiators. That is, the proposed model has better autonomy and flexibility.

Furthermore, considering the actual situation in which both agents’ proposals may be infinitely close but not necessarily equal, an ending rule is needed to reach an agreement. If the differences of the proposed values for all the attributes between the seller agent and the buyer agent are less than 1 in the l−th round, the negotiation is considered as a success and the consensus value of attribute k will be determined as the average of the seller agent’s and the buyer agent’s offers. That is,


$$P_{k}^{*}=\frac{P_{s,k}(l)+P_{b,k}(l)}{\text{2}},l=1,2,\cdots ,T$$ (12)

## 5. Experiments and analysis

In this section, we employ a comprehensive methodology, integrating numerical simulation, ablation studies, hypothesis testing, and comparative analysis, to systematically explore the influence of emotional factors and time beliefs on the automated negotiation process. First, the ablation study systematically removes the emotion and time belief components, constructing models with various combinations to assess the independent contributions of each component in improving negotiation efficiency and outcome quality. Second, sensitivity analysis is conducted to evaluate the impact of variations in key cognitive dimensions (e.g., certainty and pleasantness) and temporal parameters on model performance, ensuring the robustness and stability of the model. To further validate the findings, hypothesis testing methods, including the Friedman test and the post-hoc Nemenyi test, are introduced to assess the significance of differences between the proposed model and the baseline model. These comparisons focus on metrics such as negotiation success rate, round count, joint utility, and utility disparity, thereby validating the proposed mechanisms and supporting the research hypotheses.

In summary, this section adopts a multi-level, fine-grained experimental design, simulating a wide range of realistic negotiation scenarios within a controlled, repeatable environment. This approach not only facilitates a detailed examination of the mechanisms underlying emotional and time-related variables but also provides robust empirical evidence supporting the generalizability and applicability of the model across various practical contexts.

### 5.1 Experimental settings

Suppose that a buyer agent and a seller agent in a supply chain in the coal industry are engaged in a multi-attribute negotiation over a certain product. In coal negotiations, the price is the most important attribute, followed by the quality of coal. Since the quality of coal has multiple assessing dimensions, e.g., caloric value, ash, volatile, and sulfur content, the coal grade, a synthetic concept, is often used in the industry to comprehensively characterize the quality of coal. The data for the numerical experiments were derived from the historical transaction values of China Power Coal in Port Qinhuangdao (China Power Coal; see https://www.ceicdata.com/en/country/china), spanning from 1 May 2014–1 May 2016. The coal price varied between 300 RMB/Ton and 440 RMB/Ton with a minimum price change of 0.2 RMB/Ton. The coal grade is a categorical variable in practice, and it was converted into a continuous number defined on the interval of [20, 90] to be fed to the proposed model in the experiment.

The protocol settings and experimental design pertaining to bilateral negotiation are based on the recent study by Wu et al. [45]. Specifically, the ranges of experimental parameters are shown in Table 5, and the rules for generating initial conditions are defined as follows: (1) for the benefit-type attribute, the uniform distribution defined on the corresponding interval was employed to randomly generate the initial value (between the historical mean and maximum value), the reserved value (between the historical minimum and the mean value), and the expected value (between the reserved value and the initial value), respectively; (2) for the cost-type attribute, the uniform distribution was also employed to randomly generate the initial value (between the historical minimum and mean value), the reserved value (between the historical mean and maximum value), and the expected value (between the initial value and the reserved value), respectively.

**Table 5:** The minimum and maximum values of the buyer and seller for negotiation attributes.

Agent type	Negotiation attribute	Minimum value	Maximum value
Seller	Price	372	440
	Quality	30	55
Buyer	Price	300	372
	Quality	55	90

To ensure that the parameters are within a reasonable range and retain a certain degree of randomness, we randomly generated 1000 sets of data containing the initial value, expected value, and reservation value to simulate the persuasion process. In the real world, there are significant differences in attribute weighting between negotiating parties, which reflect subjective preferences. Here, we assume that both agents hold the same weighting preference to control this subjective term’s disturbance. Since this simulation experiment considers two negotiating attributes, there are two types of weighting relationships, i.e., the price attribute is more weighted than the quality attribute and vice versa. Considering common practice in China’s coal industry, we used the former weighting relationship and the weights of the two attributes were set to be 0.6 and 0.4 without loss of generosity. The proposed negotiation model can be easily extended to arbitrage weighting preferences.

In addition, we assume that the level of certainty of the buyer agent and that of seller agent are both high. Following the trust level classification criteria in [16], this paper adopts the same standard to divide the trust into high, middle, and low intervals (refer to Table 6), and they can accept the degree of certainty of each other. The selection rules of emotional persuasion strategies and the emotional persuasion factors are selected according to Table 3. In terms of price and quality, there is a negotiable space between the buyer and the seller, and the maximum number of negotiation rounds is considered a variable. Since this study focuses on investigating the joint effects of emotional persuasion and time belief on negotiation outcomes, we treated the maximum number of negotiation rounds as a control variable and set it to be 20. Furthermore, for the purpose of illustration, we assume that the belief density functions for both the price and quality attributes follow a uniform distribution.

**Table 6:** The classification of the degree of certainty.

Interval of the degree of certainty	Level of certainty
(0.8, 1]	High
$\begin{array}{c} \left(\text{0}.\text{5},\;\text{0}.\text{8}\right] \end{array}$	Mid
[0, 0.5]	Low

### 5.2 Evaluation metrics

Success Rate, Negotiation Rounds, Joint Utility, and Utility Difference are usually applied to measure the performance of the proposed model comprehensively [5,45,68,69]. The evaluation indicators are summarized as follows:

(1) Success Rate (SR). The negotiation success rate indicates the rate at which a negotiation eventually leads to a successful agreement. Assuming that a deal is reached n times out of N negotiation tests, the success rate is calculated as follows:


$$SR=\frac{n}{N}$$ (13)

(2) Negotiation Rounds (NR). No matter how well the negotiations work out in the end, the average number of rounds can reflect the negotiation efficiency from the time dimension. Assuming that in n automated negotiation tests, the negotiation in the i-th test takes place for ${\text{m}}_{\text{i}}$ rounds, the average number of rounds can be calculated as follow:


$$NR=\frac{\sum {\mathrm{\backslash nolimits}}_{i=1}^{n}m_{i}}{n}$$ (14)

(3) Joint Utility (JU). The joint utility measures the joint outcome of the negotiation and is the sum of the utilities obtained by both sides once an agreement is achieved:


JU=PU(b)+PU(s) (15)

where PU(b) and PU(s) stand for the buyer’s and seller’s personal utility, respectively. The personal utility can be calculated in terms of equation (1) and equation (2).

(4) Utility Difference (UD). The utility difference represents the degree of difference between the utilities of two parties, and a better outcome should be as small as possible. The utility difference can be defined as follows:


UD=|PU(b)−PU(s)| (16)

### 5.3 Experimental results and analysis of the ablation study

In this subsection, an ablation study was conducted to further verify whether the emotion and time factors considered in the proposed model play a role in improving the performance of emotional persuasion. From Section 4.5, the basic model considers neither emotion nor time, and its proposal updating formula refers to Equation (17). In addition, the proposal updating formula considering only emotion or time can be referred to Equation (18) and Equation (19), respectively. Finally, the proposal updating formula that considers both emotion and time can be referred to Equation (9).


$$\begin{array}{c} P_{i,k}(n)=\{\begin{array}{cc} {}*20lP_{i,k}(n-1)+Q_{i,k}(n), & cost-type\\ P_{i,k}(n-1)-Q_{i,k}(n), & benefit-type \end{array}\\ \;i=s\;or\;b \end{array}$$ (17)

$$\begin{array}{c} P_{i,k}(n)=\{\begin{array}{cc} {}*20lP_{i,k}(n-1)+\alpha_{i}(n)Q_{i,k}(n), & cost-type\\ P_{i,k}(n-1)-\alpha_{i}(n)Q_{i,k}(n), & benefit-type \end{array}\\ \;i=s\;or\;b \end{array}$$ (18)

$$\begin{array}{c} P_{i,k}(n)=\{\begin{array}{cc} {}*20lP_{i,k}(n-1)+f_{i}(n)Q_{i,k}(n), & cost-type\\ P_{i,k}(n-1)-f_{i}(n)Q_{i,k}(n), & benefit-type \end{array}\\ \;i=s\;or\;b \end{array}$$ (19)

where $P_{i,k}(n)$ indicates the proposal to be updated, $P_{i,k}(n-1)$ reflects the proposal in the l−th round, $Q_{i,k}(n)$ reflects the base concession range, $\alpha_{i}(n)$ represents the emotional persuasion factor, and $f_{i}(n)$ characterizes the timing-related persuasion behavior.

**Fig 3:**
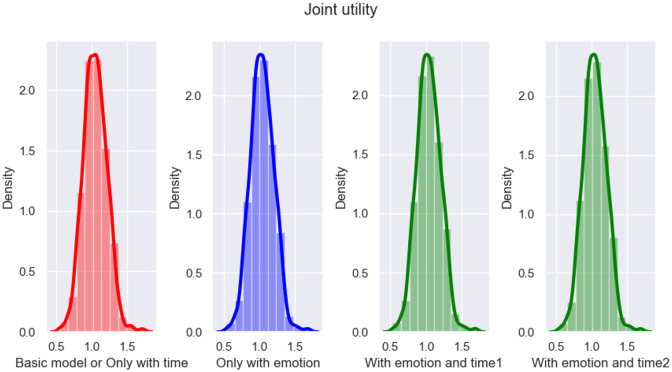
The distribution and kernel density estimation of joint utility for the ablation study results.

**Fig 4:**
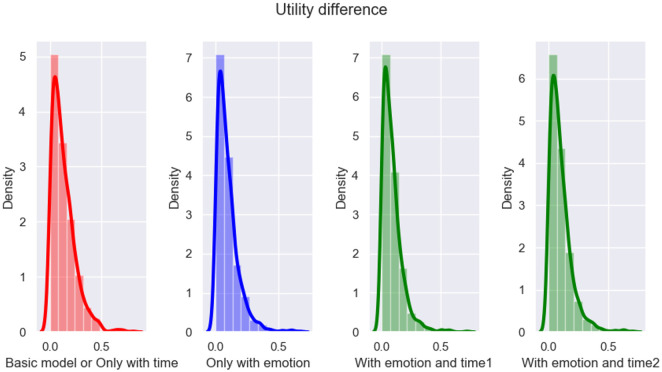
The distribution and kernel density estimation of utility difference for the ablation study results.

**Fig 5:**
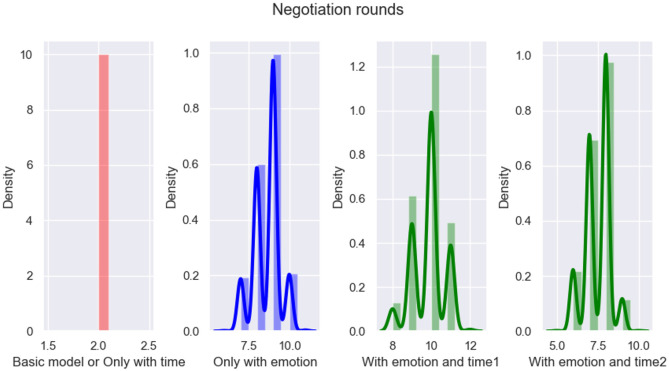
The distribution and kernel density estimation of negotiation rounds for the ablation study results.

We conducted ablation experiments on the same dataset and compared the corresponding ablative results. The experimental results are shown in Table 7. To obtain a more reliable analysis, we use the Friedman test and the Post-hoc Nemenyi test to judge whether the difference in performance between the models is significant. The tests are conducted at the 0.05 level of significance. Firstly, we use the Friedman test to evaluate whether the above models perform equally, with the null-hypothesis defined as all methods having the same performance. Then, if the null-hypothesis of the Friedman test is rejected, we further conduct the Post-hoc Nemenyi test to gain insight into the differences between the analyzed methods. The results of the Friedman test are also shown in Table 7. In addition, Figs 3–5 show the distribution and kernel density estimation of the ablation study results.

**Table 7:** Performance comparison of the ablative results.

Performance indicators	Basic model	Only with emotion	Only with time	With emotion and time1 (slow first then hurry)	With emotion and time2 (hurry first then slow)	Friedman test (p-value)
Success rate	100%	100%	100%	100%	100%	–
Joint utility	1.0499	1.0423	1.0499	1.0416	1.0435	0.00
Utility difference	0.1324	0.0946	0.1324	0.0908	0.1006	0.00
Negotiation rounds	2.0000	8.6130	2.0000	9.857	7.494	0.00

From the Friedman test in Table 7, the test results indicate that the performance of the above-mentioned experimental results differs significantly. For this reason, we further conducted the Post-hoc Nemenyi test. The results of the Post-hoc Nemenyi test are shown in Table 8, which indicates that only the performance of the basic model and the model considering only the time factor is the same, and there is a significant difference between the performance of the other groups.

**Table 8:** The result of the Post-hoc Nemenyi test for the ablation study results.

	Post-hoc Nemenyi test (p-value)				
**Negotiation rounds**	Basic model	Only with emotion	Only with time	With emotion and time1	With emotion and time2
Basic model	1.00	$\text{1}.\text{05}\times {\text{10}}^{-\text{246}}$	1.00	0.00	$\text{7}.\text{41}\times {\text{10}}^{-\text{103}}$
Only with emotion	$\text{1}.\text{05}\times {\text{10}}^{-\text{246}}$	1.00	$\text{1}.\text{05}\times {\text{10}}^{-\text{246}}$	$\text{1}.\text{02}\times {\text{10}}^{-\text{47}}$	$\text{8}.\text{33}\times {\text{10}}^{-\text{43}}$
Only with time	1.00	$\text{1}.\text{05}\times {\text{10}}^{-\text{246}}$	1.00	0.00	$\text{7}.\text{41}\times {\text{10}}^{-\text{103}}$
With emotion and time1	0.00	$\text{1}.\text{02}\times {\text{10}}^{-\text{47}}$	0.00	1.00	$\text{1}.\text{13}\times {\text{10}}^{-\text{163}}$
With emotion and time2	$\text{7}.\text{41}\times {\text{10}}^{-\text{103}}$	$\text{8}.\text{33}\times {\text{10}}^{-\text{43}}$	$\text{7}.\text{41}\times {\text{10}}^{-\text{103}}$	$\text{1}.\text{13}\times {\text{10}}^{-\text{163}}$	1.00
**Joint utility**	Basic model	Only with emotion	Only with time	With emotion and time1	With emotion and time2
Basic model	1.00	$\text{4}.\text{81}\times {\text{10}}^{-\text{189}}$	1.00	$\text{4}.\text{81}\times {\text{10}}^{-\text{189}}$	$\text{4}.\text{81}\times {\text{10}}^{-\text{189}}$
Only with emotion	$\text{4}.\text{81}\times {\text{10}}^{-\text{189}}$	1.00	$\text{4}.\text{81}\times {\text{10}}^{-\text{189}}$	$\text{2}.\text{00}\times {\text{10}}^{-\text{3}}$	$\text{9}.\text{32}\times {\text{10}}^{-\text{15}}$
Only with time	1.00	$\text{4}.\text{81}\times {\text{10}}^{-\text{189}}$	1.00	$\text{1}.\text{31}\times {\text{10}}^{-\text{223}}$	$\text{7}.\text{97}\times {\text{10}}^{-\text{112}}$
With emotion and time1	$\text{4}.\text{81}\times {\text{10}}^{-\text{189}}$	$\text{2}.\text{00}\times {\text{10}}^{-\text{3}}$	$\text{1}.\text{31}\times {\text{10}}^{-\text{223}}$	1.00	$\text{3}.\text{80}\times {\text{10}}^{-\text{27}}$
With emotion and time2	$\text{4}.\text{81}\times {\text{10}}^{-\text{189}}$	$\text{9}.\text{32}\times {\text{10}}^{-\text{15}}$	$\text{7}.\text{97}\times {\text{10}}^{-\text{112}}$	$\text{3}.\text{80}\times {\text{10}}^{-\text{27}}$	1.00
Utility difference	Basic model	Only with emotion	Only with time	With emotion and time1	With emotion and time2
Basic model	1.00	$\text{3}.\text{32}\times {\text{10}}^{-\text{177}}$	1.00	$\text{1}.\text{50}\times {\text{10}}^{-\text{207}}$	$\text{2}.\text{79}\times {\text{10}}^{-\text{106}}$
Only with emotion	$\text{3}.\text{32}\times {\text{10}}^{-\text{177}}$	1.00	$\text{3}.\text{32}\times {\text{10}}^{-\text{177}}$	$\text{5}.\text{47}\times {\text{10}}^{-\text{3}}$	$\text{3}.\text{32}\times {\text{10}}^{-\text{13}}$
Only with time	1.00	$\text{3}.\text{32}\times {\text{10}}^{-\text{177}}$	1.00	$\text{1}.\text{50}\times {\text{10}}^{-\text{207}}$	$\text{2}.\text{79}\times {\text{10}}^{-\text{106}}$
With emotion and time1	$\text{1}.\text{50}\times {\text{10}}^{-\text{207}}$	$\text{5}.\text{47}\times {\text{10}}^{-\text{3}}$	$\text{1}.\text{50}\times {\text{10}}^{-\text{207}}$	1.00	$\text{1}.\text{24}\times {\text{10}}^{-\text{23}}$
With emotion and time2	$\text{2}.\text{79}\times {\text{10}}^{-\text{106}}$	$\text{3}.\text{32}\times {\text{10}}^{-\text{13}}$	$\text{2}.\text{79}\times {\text{10}}^{-\text{106}}$	$\text{1}.\text{24}\times {\text{10}}^{-\text{23}}$	1.00

On the one hand, by comparing the results of the basic model and the model with only emotion added, it is clear that the utility difference between buyers and sellers can be reduced by 28.55% significantly when considering emotion as an influencing factor in this paper. In addition, as we can see, the basic method completes the negotiation almost at the beginning of the negotiation, which is unreasonable [67]. This is because a certain amount of time and communication is required to avoid excessive concessions and ensure that both parties have a clear understanding of each other’s views and intentions. Adding emotion can make the pace of negotiations correspond to the reality of business negotiations [70]. In short, it is of significance to consider emotion as an influencing factor for improving negotiation performance.

On the other hand, when we directly compare the basic model with the model that considers only the time factor, we find that the time factor seems to have no effect on the negotiation performance. This is because the basic model compromises too quickly, and adding the time factor directly to the basic model does not work, which indirectly indicates the poor scalability of the basic model. Therefore, in this paper, the time factor is considered after adding the emotion factor to make the negotiation speed reasonable. In addition, the above results show that the time function of “fast first then slow” proposed in this paper can significantly reduce the number of emotional persuasion negotiation rounds and improve the negotiation efficiency by 12.99% on the basis of guaranteeing other negotiation performance. At the same time, the experimental results can also show that considering individuals’ time behavior on the basis of emotional persuasion can further satisfy individuals’ expectations of the length of negotiation while ensuring the success rate of negotiation [10]. For example, individuals with greater time pressure tend to finish the negotiation as early as possible, while individuals with less time pressure are usually willing to spend more time on the negotiation.

The experimental results show that both emotional and temporal factors, when acting independently, can significantly improve negotiation performance. The introduction of emotional factors leads to varying degrees of improvement in fairness and success rate, while the introduction of temporal factors enhances negotiation efficiency and keeps other performance metrics stable. This indicates that the main effects of the two factors are prominent and can each make a substantial contribution to negotiation performance without relying on interaction terms. This finding also supports the rationale for our independent modeling approach.

In summary, the model proposed in this paper can effectively reduce the utility difference through the emotional persuasion process compared to the basic model. Meanwhile, the problem of increasing negotiation rounds caused by emotional persuasion can be further corrected by adding the time function. In short, emotional persuasion can not only improve the fairness of the negotiation but also make the negotiation speed more reasonable, and further consideration of the time function can be appropriate to speed up the negotiation on a reasonable basis, as well as optimize other performance.

### 5.4 Analysis of persuasion results under different emotions

In this section, a simulation experiment is conducted to evaluate the specific emotional strategy, with the objective of verifying the validity of the deconstruction of emotions into cognitive dimensions and their subsequent mapping to persuasion strategies. Specifically, six discrete emotions—anger, disappointment, anxiety, anticipation, gratification, and joy—are assigned to the agents of both negotiating parties. The negotiation attributes remain constant, and each emotion is associated with distinct levels of pleasantness, certainty, and mappings to the strategies listed in Table 3. Additionally, a baseline group is established using the base model without emotion as a control, allowing for a comparison with the emotion group to assess the influence of different emotions on negotiation outcomes.

**Fig 6:**
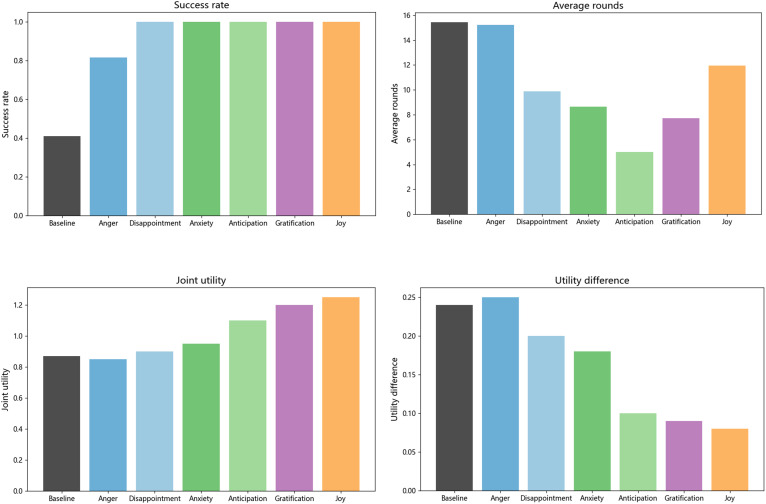
Comparison of negotiation outcomes between the baseline and emotion-specific persuasion methods.

The parameters used in this experiment were consistent with those employed in the ablation experiments, with attributes such as attribute intervals and attribute weights held constant. The persuasion strategy for each emotional state was determined according to the mapping relationship presented in Table 3. A total of 1,000 experiments were conducted for each emotional state group to observe changes in negotiation success rate, number of negotiation rounds, joint utility, and utility difference. The mean values for each experimental metric are presented in bar charts, with specific results displayed in Fig 6.

As shown in Fig 6, the two cognitive dimensions of emotion—pleasantness and certainty—within the ATF framework developed in this study systematically influence negotiators’ concession strategies. Specifically, negotiators experiencing anger (low pleasantness, high certainty) tend to adopt more aggressive concession strategies. These negotiations are generally prolonged (averaging 15.2 rounds), exhibit a slightly lower success rate (approximately 81.7%), and result in suboptimal agreement quality (joint utility of 0.85, with a utility difference of 0.25). This suggests that negative emotions, when combined with high certainty, reduce negotiators’ willingness to concede, thus hindering cooperation. In contrast, negotiators experiencing anticipation (moderate pleasantness, moderate certainty) reach agreements more quickly, with a success rate approaching 100% in just 5 rounds, yielding the highest joint utility (1.25) and the most equitable distribution of benefits (utility difference of 0.08). This indicates that positive emotional states with moderate certainty facilitate more efficient reciprocal concession strategies and enhance the overall quality of negotiation outcomes. Similarly, the group experiencing joy (high pleasantness, high certainty) achieved a 100% success rate and a significantly higher joint utility (mean rounds: 11.9), outperforming the no-emotion baseline group (which had a success rate of only 41%). Although the negotiation process for the joy group was slightly longer than that of the anticipation group, the marked differences in success rate, negotiation efficiency, and outcome fairness highlight the crucial role of emotional factors in shaping negotiation decisions.

As reflected in the experimental results, not all modeled emotions have a significant positive impact on negotiation outcomes. While certain emotions (such as happiness, excitement, and, in specific contexts, anger) can enhance joint utility, fairness, or persuasion success rate, other emotions (such as fear and sadness) show less consistent contributions. In this study, our decision to include the full range of emotions is based on two main considerations: first, we aim to comprehensively validate the theoretical applicability of the ATF in automated negotiation. To examine the effectiveness of this theoretical framework across various emotion types, it is necessary to encompass the performance and mechanisms of different emotions, even if their positive effects are not significant. Second, from the perspective of full-spectrum agent modeling and interpretability, retaining the complete set of emotions enables the negotiation agent to exhibit richer and more realistic emotional response patterns during interactions, thereby enhancing its ability to simulate real negotiation scenarios and providing a solid experimental foundation for applicability in future human–agent settings.

In summary, the above findings demonstrate that the emotional persuasion strategy, based on the ATF framework and incorporating the dimensions of pleasantness and certainty, effectively improves negotiation outcomes by guiding concession strategies based on emotional analysis.

### 5.5 Sensitivity analysis

#### 5.5.1 Sensitivity analysis of the initial trustworthiness.

The trustworthiness levels of buyers and sellers may be different before negotiation, and the trust levels of negotiating parties are the basis for constructing the cognitive dimension of certainty. Therefore, in order to further analyze the effect of the trust level of negotiation parties on the negotiation outcome, this subsection conducted experiments on the given data set with parties at different trust levels. The scenarios are shown in Table 9. The experimental results are shown in Table 10.

**Table 10:** The results under different scenarios of the negotiating parties’ initial trustworthiness.

Scenarios	Performance indicators			
	Buyer’s utility	Seller’s utility	Joint utility	Utility difference
1	0.5263	0.5154	1.0417	0.0908
2	0.5242	0.5184	1.0425	0.0951
3	0.6639	0.3865	1.0504	0.2843
4	0.5285	0.5137	1.0421	0.0935
5	0.5263	0.5167	1.0430	0.0975
6	0.6758	0.3769	1.0527	0.3053
7	0.3927	0.6495	1.0423	0.2615
8	0.3828	0.6618	1.0447	0.2836
9	0.5315	0.5225	1.0539	0.1521

**Table 9:** Different scenarios of the negotiating parties’ initial trustworthiness.

Scenarios	The buyer’s trustworthiness	The seller’s trustworthiness
1	(0.8, 1]	(0.8, 1]
2	(0.8, 1]	(0.6, 0.8]
3	(0.8, 1]	[0, 0.6]
4	(0.6, 0.8]	(0.8, 1]
5	(0.6, 0.8]	(0.6, 0.8]
6	(0.6, 0.8]	[0, 0.6]
7	[0, 0.6]	(0.8, 1]
8	[0, 0.6]	(0.6, 0.8]
9	[0, 0.6]	[0, 0.6]

The success rate of negotiation was 100% in the above nine scenarios. By fixing the trust level of one party and adjusting the trust level of the other party, the results of individual utility, joint utility, and utility difference between the negotiating buyer and seller were obtained as shown in Table 10. The above results show that when the party with a higher trust level negotiates with the party with a lower trust level, the party with a higher trust level can obtain a higher individual utility. Moreover, as the gap between the two parties’ trust levels increases, the higher trust level gains higher individual utility, which directly leads to an increase in the utility difference and indirectly leads to an increase in the joint utility.

**Fig 7:**
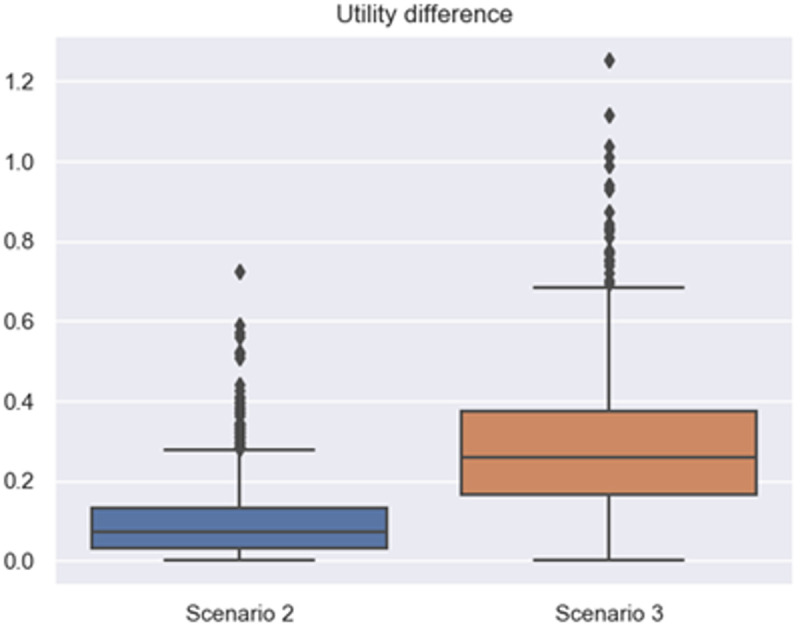
Comparison of utility differences between negotiating parties under scenarios 2 and 3.

In addition, we take scenarios 2 and 3 as examples, and Fig 7 shows the utility differences between the negotiating parties in the two scenarios. As shown by Fig 7, the overall utility difference of scenario 2 is significantly smaller than the overall utility difference of scenario 3.

#### 5.5.2 Sensitivity analysis of the initial cognitive dimensions in the ATF.

This paper considers two core cognitive dimensions of agents based on the theory of ATF, namely certainty and pleasantness. Therefore, we conduct experiments by combining these two cognitive dimensions at different levels. As it is known in Section 3, the certainty dimension is represented by 3 levels (high, mid, and low), while the pleasure dimension is determined by the agent’s perception coefficient km. Here, we make km equal to 0.5, 1, and 1.5, respectively. The results of the combinations are shown in Table 11. The experimental results are shown in Table 12.

**Table 11:** Different combinations of the cognitive dimensions in the ATF.

Combinations	The level of certainty	the coefficient of pleasantness (km)
1	High	1.5
2	High	1
3	High	0.5
4	Mid	1.5
5	Mid	1
6	Mid	0.5
7	Low	1.5
8	Low	1
9	Low	0.5

**Table 12:** The results under different combinations of the cognitive dimensions in the ATF.

Combinations		Performance indicators			
	Negotiation rounds	Buyer’s utility	Seller’s utility	Joint utility	Utility difference
1	9.375	0.5274	0.5158	1.0432	0.0973
2	9.857	0.5263	0.5154	1.0417	0.0908
3	10.534	0.5240	0.5156	1.0397	0.0828
4	8.938	0.5274	0.5173	1.0446	0.1043
5	9.447	0.5263	0.5167	1.0430	0.0975
6	10.114	0.5240	0.5169	1.0408	0.0887
7	13.768	0.5315	0.5223	1.0539	0.1519
8	13.768	0.5315	0.5225	1.0539	0.1521
9	13.768	0.5315	0.5225	1.0539	0.1523

The negotiation success rate was 100% for all the different combinations above. The above results show that when the pleasantness coefficient is constant, as the certainty level decreases, the individual utility, joint utility, and utility difference between buyers and sellers tend to increase, and the number of negotiation rounds fluctuates. When the certainty level is fixed and the certainty level is medium or high, the negotiation rounds gradually increases as the pleasantness coefficient decreases, and the joint utility and utility difference tend to decrease; when the certainty level is low, the utility difference of the negotiation results is affected by the change of the pleasantness dimension.

In summary, we can conclude that both certainty and pleasantness have a significant impact on negotiation results in the Appraisal Tendency Framework, and there is an interaction between these two dimensions.

### 5.6 Comparisons

#### 5.6.1 Comparison with the fixed discount parameter method.

This section compares the proposed concession updating algorithm, which is based on the variable parameter $f_{i}(n)$, with the methods that use a constant time discount factor denoted as λ. The numerical experiment is conducted with the same parameter settings.

With the same parameters set in the numerical experiment, this section compares the proposed concession updating algorithm based on variable parameter $f_{i}(n)$ with the method which has a constant discount factor denoted as λ. The experimental results and Friedman test results are shown in Table 13.

**Table 13:** Comparison between the proposed method and the fixed discount parameter method.

	Methods with fixed discount parameter				The proposed model		Friedman test (p-value)
**Performance** **indicators**	λ1=0.3	λ2=0.5		λ3=0.7	λ5=0.9	$f_{i}\text{1}(\text{n})=\text{ln}(\text{e}+\frac{n}{T}-\text{0}.\text{5})$	$f_{i}\text{2}(\text{n})=\text{ln}(\text{e}+\text{0}.\text{5}-\frac{n}{T})$
Success rate	0%	88%	**100%**	**100%**	**100%**	**100%**	–
Joint utility	1.0398	1.0404	1.0411	1.0419	1.0416	**1.0435**	$\text{1}.\text{65}\times {\text{10}}^{-\text{172}}$
Utility difference	**0.0801**	0.0836	0.0878	0.0920	0.0908	0.1006	$\text{9}.\text{27}\times {\text{10}}^{-\text{179}}$
Negotiation rounds	32.125	18.798	13.019	9.769	9.857	**7.494**	0.00

Note: the better value of each indicator is bolded.

The results of the Friedman test show that the performance of the experimental results differs significantly among the analyzed methods, so we further conducted the Post-hoc Nemenyi test. The results of the Post-hoc Nemenyi test are shown in Table 14.

**Table 14:** The result of the Post-hoc Nemenyi test for different methods.

	Post-hoc Nemenyi test (p-value)					
**Negotiation rounds**	λ1	λ2	λ3	λ4	$f_{i}\text{1}(n)$	$f_{i}\text{2}(n)$
λ1	1.00	$\text{3}.\text{50}\times {\text{10}}^{-\text{33}}$	$\text{3}.\text{14}\times {\text{10}}^{-\text{122}}$	0.00	0.00	0.00
λ2	$\text{3}.\text{50}\times {\text{10}}^{-\text{33}}$	1.00	$\text{3}.\text{49}\times {\text{10}}^{-\text{33}}$	$\text{2}.\text{29}\times {\text{10}}^{-\text{190}}$	$\text{1}.\text{50}\times {\text{10}}^{-\text{178}}$	0.00
λ3	$\text{3}.\text{14}\times {\text{10}}^{-\text{122}}$	$\text{3}.\text{49}\times {\text{10}}^{-\text{33}}$	1.00	$\text{3}.\text{25}\times {\text{10}}^{-\text{75}}$	$\text{2}.\text{49}\times {\text{10}}^{-\text{67}}$	$\text{7}.\text{25}\times {\text{10}}^{-\text{256}}$
λ4	0.00	$\text{2}.\text{29}\times {\text{10}}^{-\text{190}}$	$\text{3}.\text{25}\times {\text{10}}^{-\text{75}}$	1.00	$\text{2}.\text{87}\times {\text{10}}^{-\text{1}}$	$\text{2}.\text{49}\times {\text{10}}^{-\text{67}}$
$f_{i}\text{1}(n)$	0.00	$\text{1}.\text{50}\times {\text{10}}^{-\text{178}}$	$\text{2}.\text{49}\times {\text{10}}^{-\text{67}}$	$\text{2}.\text{87}\times {\text{10}}^{-\text{1}}$	1.00	$\text{3}.\text{25}\times {\text{10}}^{-\text{75}}$
$f_{i}\text{2}(n)$	0.00	0.00	$\text{7}.\text{25}\times {\text{10}}^{-\text{256}}$	$\text{2}.\text{49}\times {\text{10}}^{-\text{67}}$	$\text{3}.\text{25}\times {\text{10}}^{-\text{75}}$	1.00
**Joint utility**	λ1	λ2	λ3	λ4	$f_{i}\text{1}(n)$	$f_{i}\text{2}(n)$
λ1	1.00	$\text{1}.\text{36}\times {\text{10}}^{-\text{6}}$	$\text{1}.\text{89}\times {\text{10}}^{-\text{30}}$	$\text{3}.\text{59}\times {\text{10}}^{-\text{72}}$	$\text{4}.\text{49}\times {\text{10}}^{-\text{32}}$	$\text{3}.\text{28}\times {\text{10}}^{-\text{130}}$
λ2	$\text{1}.\text{36}\times {\text{10}}^{-\text{6}}$	1.00	$\text{2}.\text{17}\times {\text{10}}^{-\text{11}}$	$\text{2}.\text{15}\times {\text{10}}^{-\text{40}}$	$\text{2}.\text{26}\times {\text{10}}^{-\text{12}}$	$\text{4}.\text{15}\times {\text{10}}^{-\text{87}}$
λ3	$\text{1}.\text{89}\times {\text{10}}^{-\text{30}}$	$\text{3}.\text{28}\times {\text{10}}^{-\text{130}}$	1.00	$\text{2}.\text{08}\times {\text{10}}^{-\text{11}}$	$\text{7}.\text{45}\times {\text{10}}^{-\text{1}}$	$\text{1}.\text{21}\times {\text{10}}^{-\text{40}}$
λ4	$\text{3}.\text{59}\times {\text{10}}^{-\text{72}}$	$\text{2}.\text{15}\times {\text{10}}^{-\text{40}}$	$\text{2}.\text{08}\times {\text{10}}^{-\text{11}}$	1.00	$\text{1}.\text{81}\times {\text{10}}^{-\text{10}}$	$\text{1}.\text{61}\times {\text{10}}^{-\text{11}}$
$f_{i}\text{1}(n)$	$\text{3}.\text{59}\times {\text{10}}^{-\text{72}}$	$\text{2}.\text{26}\times {\text{10}}^{-\text{12}}$	$\text{7}.\text{45}\times {\text{10}}^{-\text{1}}$	$\text{1}.\text{81}\times {\text{10}}^{-\text{10}}$	1.00	$\text{8}.\text{16}\times {\text{10}}^{-\text{39}}$
$f_{i}\text{2}(n)$	$\text{3}.\text{28}\times {\text{10}}^{-\text{130}}$	$\text{4}.\text{15}\times {\text{10}}^{-\text{87}}$	$\text{1}.\text{21}\times {\text{10}}^{-\text{40}}$	$\text{1}.\text{61}\times {\text{10}}^{-\text{11}}$	$\text{8}.\text{16}\times {\text{10}}^{-\text{39}}$	1.00
**Utility difference**	λ1	λ2	λ3	λ4	$f_{i}\text{1}(n)$	$f_{i}\text{2}(n)$
λ1	1.00	$\text{6}.\text{27}\times {\text{10}}^{-\text{7}}$	$\text{8}.\text{11}\times {\text{10}}^{-\text{33}}$	$\text{1}.\text{46}\times {\text{10}}^{-\text{78}}$	$\text{1}.\text{37}\times {\text{10}}^{-\text{39}}$	$\text{4}.\text{49}\times {\text{10}}^{-\text{132}}$
λ2	$\text{6}.\text{27}\times {\text{10}}^{-\text{7}}$	1.00	$\text{2}.\text{37}\times {\text{10}}^{-\text{12}}$	$\text{2}.\text{25}\times {\text{10}}^{-\text{44}}$	$\text{1}.\text{41}\times {\text{10}}^{-\text{16}}$	$\text{2}.\text{00}\times {\text{10}}^{-\text{87}}$
λ3	$\text{8}.\text{11}\times {\text{10}}^{-\text{33}}$	$\text{2}.\text{37}\times {\text{10}}^{-\text{12}}$	1.00	$\text{1}.\text{59}\times {\text{10}}^{-\text{12}}$	$\text{2}.\text{06}\times {\text{10}}^{-\text{1}}$	$\text{4}.\text{54}\times {\text{10}}^{-\text{39}}$
λ4	$\text{1}.\text{46}\times {\text{10}}^{-\text{78}}$	$\text{2}.\text{25}\times {\text{10}}^{-\text{44}}$	$\text{1}.\text{59}\times {\text{10}}^{-\text{12}}$	1.00	$\text{6}.\text{26}\times {\text{10}}^{-\text{9}}$	$\text{1}.\text{11}\times {\text{10}}^{-\text{9}}$
$f_{i}\text{1}(n)$	$\text{1}.\text{37}\times {\text{10}}^{-\text{39}}$	$\text{1}.\text{41}\times {\text{10}}^{-\text{16}}$	$\text{2}.\text{06}\times {\text{10}}^{-\text{1}}$	$\text{6}.\text{26}\times {\text{10}}^{-\text{9}}$	1.00	$\text{2}.\text{41}\times {\text{10}}^{-\text{32}}$
$f_{i}\text{2}(n)$	$\text{4}.\text{49}\times {\text{10}}^{-\text{132}}$	$\text{2}.\text{00}\times {\text{10}}^{-\text{87}}$	$\text{2}.\text{06}\times {\text{10}}^{-\text{1}}$	$\text{1}.\text{11}\times {\text{10}}^{-\text{9}}$	$\text{2}.\text{41}\times {\text{10}}^{-\text{32}}$	1.00

The above results show that there is no significant difference between the method with parameter $f_{i}1(n)$ and the method with parameter λ4 in the indicator of the negotiation round; meanwhile, there is no significant difference between the method with parameter $f_{i}1(n)$ and the method with parameter λ3 in the utility-related indicators; except for these, the performance difference of other comparison groups is significant. Most obviously, the method with the fixed parameter requires more rounds of emotional persuasion to reach an agreement, which takes more turns and also leads to a lower success rate. Therefore, it is necessary to select an appropriate value of λ to apply the basic method with a fixed parameter.

When λ is small, both buyer agent and seller agent can reduce their concession ranges, but more rounds of persuasion are required to reach an agreement. In such a situation, the emotional persuasion may fail due to the limit of the maximum number of rounds of persuasion. When λ is large, both agents will increase their concession ranges, so that the emotional persuasion will reach an agreement as soon as possible. During this process, however, one agent’s interest may be seriously damaged because of the large concession range resulting from a large λ. The model proposed in this paper can make an agent choose a dynamic concession range according to its own attitude towards time; as such, a reasonable persuasion result can be obtained without considering how to set a suitable discount factor.

#### 5.6.2 Comparison with on other time-belief functions.

In the references [29,30], they both proposed a time-belief function to improve the negotiation process. The belief function (hereafter referred to as bfs) in literature [30] takes the form of a monotonic decreasing function (i.e., bfs=1−t/T), and the belief function in literature [29] is a monotonic increasing function (i.e., bfs=t/T).

Table 15 compares the negotiation results and Friedman test results using the above method based on a time-belief function with the model established by this paper. Table 16 presents the post-hoc Nemenyi test results for different emotional persuasion based on time belief.

**Table 15:** Comparison of negotiation results between the proposed method and the other method based on time belief.

	The other method based on time belief		The proposed model		Friedman test (p-value)
**Performance** **indicators**	bfs1=t/T	bfs2=1−t/T	$f_{i}\text{1}(n)=\text{ln}(\text{e}+\frac{\text{n}}{\text{T}}-\text{0}.\text{5})$	$f_{i}\text{2}(n)=\text{ln}(\text{e}+\text{0}.\text{5}-\frac{n}{T})$	
Success rate	95.6%	**100%**	**100%**	**100%**	–
Joint utility	1.0396	1.0430	1.0416	**1.0435**	$\text{5}.\text{75}\times {\text{10}}^{-\text{96}}$
Utility difference	**0.0790**	0.1006	0.0908	0.1006	$\text{8}.\text{84}\times {\text{10}}^{-\text{102}}$
Negotiation rounds	19.349	11.9	9.857	**7.494**	0.00

Note: the better value of each indicator is bolded.

**Table 16:** The result of the Post-hoc Nemenyi test for different emotional persuasion based on time belief.

Post-hoc Nemenyi test (p-value)				
**Negotiation rounds**	bfs1	bfs2	$f_{i}\text{1}(n)$	$f_{i}\text{2}(n)$
bfs1	1.00	$\text{1}.\text{91}\times {\text{10}}^{-\text{107}}$	$\text{5}.\text{06}\times {\text{10}}^{-\text{185}}$	0.00
bfs2	$\text{1}.\text{91}\times {\text{10}}^{-\text{107}}$	1.00	$\text{2}.\text{74}\times {\text{10}}^{-\text{16}}$	$\text{2}.\text{73}\times {\text{10}}^{-\text{148}}$
$f_{i}\text{1}(n)$	$\text{5}.\text{06}\times {\text{10}}^{-\text{185}}$	$\text{2}.\text{74}\times {\text{10}}^{-\text{16}}$	1.00	$\text{8}.\text{18}\times {\text{10}}^{-\text{78}}$
$f_{i}\text{2}(n)$	0.00	$\text{2}.\text{73}\times {\text{10}}^{-\text{148}}$	$\text{8}.\text{18}\times {\text{10}}^{-\text{78}}$	1.00
**Joint utility**	bfs1	bfs2	$f_{i}\text{1}(n)$	$f_{i}\text{2}(n)$
bfs1	1.00	$\text{4}.\text{44}\times {\text{10}}^{-\text{55}}$	$\text{1}.\text{96}\times {\text{10}}^{-\text{22}}$	$\text{2}.\text{48}\times {\text{10}}^{-\text{81}}$
bfs2	$\text{4}.\text{44}\times {\text{10}}^{-\text{55}}$	1.00	$\text{9}.\text{66}\times {\text{10}}^{-\text{10}}$	$\text{1}.\text{86}\times {\text{10}}^{-\text{4}}$
$f_{i}\text{1}(n)$	$\text{1}.\text{96}\times {\text{10}}^{-\text{22}}$	$\text{9}.\text{66}\times {\text{10}}^{-\text{10}}$	1.00	$\text{1}.\text{16}\times {\text{10}}^{-\text{22}}$
$f_{i}\text{2}(n)$	$\text{2}.\text{48}\times {\text{10}}^{-\text{81}}$	$\text{1}.\text{86}\times {\text{10}}^{-\text{4}}$	$\text{1}.\text{16}\times {\text{10}}^{-\text{22}}$	1.00
**Utility difference**	bfs1	bfs2	$f_{i}\text{1}(n)$	$f_{i}\text{2}(n)$
bfs1	1.00	$\text{1}.\text{13}\times {\text{10}}^{-\text{63}}$	$\text{8}.\text{17}\times {\text{10}}^{-\text{28}}$	$\text{3}.\text{84}\times {\text{10}}^{-\text{83}}$
bfs2	$\text{1}.\text{13}\times {\text{10}}^{-\text{63}}$	1.00	$\text{5}.\text{97}\times {\text{10}}^{-\text{10}}$	$\text{7}.\text{34}\times {\text{10}}^{-\text{6}}$
$f_{i}\text{1}(n)$	$\text{8}.\text{17}\times {\text{10}}^{-\text{28}}$	$\text{5}.\text{97}\times {\text{10}}^{-\text{10}}$	1.00	$\text{9}.\text{96}\times {\text{10}}^{-\text{19}}$
$f_{i}\text{2}(n)$	$\text{3}.\text{84}\times {\text{10}}^{-\text{83}}$	$\text{7}.\text{34}\times {\text{10}}^{-\text{6}}$	$\text{9}.\text{96}\times {\text{10}}^{-\text{19}}$	1.00

The Post-hoc Nemenyi test was also conducted after the results of the Friedman test showed that the performance of the analyzed methods differed significantly. The above results and analysis show that the comprehensive performance of the proposed method using the time function in this paper is significantly better than that of the method using the above-mentioned time belief functions, especially in terms of negotiation efficiency. A further analysis demonstrates that the time belief function always reduces concession ranges, but cannot reflect the acceleration effects of time on concession ranges. Therefore, a continuous reduction in concession ranges slows down the convergence rate of emotional persuasion, so that an agreement may not be likely to be reached when the persuasion arrives at the maximum round. The time function established in this paper can speed up or slow down the process of emotional persuasion, which is conducive to the success of emotional persuasion.

#### 5.6.3 Comparisons with other existing competing methods.

In order to further prove the validity of the proposed model, we compared it with other existing competing methods, which also aim to improve the intelligence of the agent.

- The portfolio strategy persuasion model [5] integrates negotiation strategies such as time-dependent and behavior-dependent.
- The hybrid strategy model [6] takes into account the opponent’s behavioral changes and remaining time as well as the opponent’s emotional state.
- The emotional persuasion model [7] constructs an emotional agent that integrates emotional rendering and reasoning capabilities to select persuasion strategies and update negotiation proposals.

We conducted experiments for the above methods on the same dataset. The results were compared with the model proposed in this paper regarding the indicators of negotiation success rate, joint utility, utility difference, and negotiation rounds. To obtain a more reliable analysis, we use the Friedman test and the Post-hoc Nemenyi test to judge whether the difference in performance between the models is significant. The experimental results with the Friedman test are shown in Table 17. Meanwhile, the results of the Post-hoc Nemenyi test are shown in Table 18, and the test results indicate that the performance of different methods is significantly different.

**Table 17:** Comparison of negotiation results between the proposed method and the most up-to-date methods.

Performance indicators	Reference [5]	Reference [6]	Reference [7]	The proposed method	Friedman test (p-value)
Success rate	60%	**100%**	**100%**	**100%**	–
Negotiation rounds	77.971	2.036	13.679	**7.494**	0.0
Joint utility	**1.2294**	1.0202	0.9901	1.0435	$\text{7}.\text{36}\times {\text{10}}^{-\text{141}}$
Utility difference	0.3252	0.4088	**0.0480**	0.1006	$\text{6}.\text{65}\times {\text{10}}^{-\text{301}}$

Note: the better value of each indicator is bolded.

**Table 18:** The result of the Post-hoc Nemenyi test for different models.

Post-hoc Nemenyi test (p-value)				
**Negotiation rounds**	Reference [5]	Reference [6]	Reference [7]	The proposed method
Reference [5]	1.00	0.00	$\text{4}.\text{09}\times {\text{10}}^{-\text{64}}$	$\text{2}.\text{10}\times {\text{10}}^{-\text{221}}$
Reference [6]	0.00	1.00	$\text{2}.\text{10}\times {\text{10}}^{-\text{221}}$	$\text{4}.\text{09}\times {\text{10}}^{-\text{64}}$
Reference [7]	$\text{4}.\text{09}\times {\text{10}}^{-\text{64}}$	$\text{2}.\text{10}\times {\text{10}}^{-\text{221}}$	1.00	$\text{4}.\text{09}\times {\text{10}}^{-\text{64}}$
The proposed method	$\text{2}.\text{10}\times {\text{10}}^{-\text{221}}$	$\text{4}.\text{09}\times {\text{10}}^{-\text{64}}$	$\text{4}.\text{09}\times {\text{10}}^{-\text{64}}$	1.00
**Joint utility**	Reference [5]	Reference [6]	Reference [7]	The proposed method
Reference [5]	1.00	$\text{2}.\text{88}\times {\text{10}}^{-\text{26}}$	$\text{2}.\text{87}\times {\text{10}}^{-\text{47}}$	$\text{4}.\text{56}\times {\text{10}}^{-\text{2}}$
Reference [6]	$\text{2}.\text{88}\times {\text{10}}^{-\text{26}}$	1.00	$\text{4}.\text{43}\times {\text{10}}^{-\text{129}}$	$\text{4}.\text{65}\times {\text{10}}^{-\text{36}}$
Reference [7]	$\text{2}.\text{87}\times {\text{10}}^{-\text{47}}$	$\text{4}.\text{43}\times {\text{10}}^{-\text{129}}$	1.00	$\text{5}.\text{16}\times {\text{10}}^{-\text{36}}$
The proposed method	$\text{4}.\text{56}\times {\text{10}}^{-\text{2}}$	$\text{4}.\text{65}\times {\text{10}}^{-\text{36}}$	$\text{5}.\text{16}\times {\text{10}}^{-\text{36}}$	1.00
**Utility difference**	Reference [5]	Reference [6]	Reference [7]	The proposed method
Reference [5]	1.00	$\text{5}.\text{01}\times {\text{10}}^{-\text{14}}$	$\text{5}.\text{48}\times {\text{10}}^{-\text{140}}$	$\text{1}.\text{39}\times {\text{10}}^{-\text{46}}$
Reference [6]	$\text{5}.\text{01}\times {\text{10}}^{-\text{14}}$	1.00	$\text{3}.\text{31}\times {\text{10}}^{-\text{216}}$	$\text{8}.\text{35}\times {\text{10}}^{-\text{101}}$
Reference [7]	$\text{5}.\text{48}\times {\text{10}}^{-\text{140}}$	$\text{3}.\text{31}\times {\text{10}}^{-\text{216}}$	1.00	$\text{1}.\text{91}\times {\text{10}}^{-\text{32}}$
The proposed method	$\text{1}.\text{39}\times {\text{10}}^{-\text{46}}$	$\text{8}.\text{35}\times {\text{10}}^{-\text{101}}$	$\text{1}.\text{91}\times {\text{10}}^{-\text{32}}$	1.00

The negotiation success rate and the average number of negotiation rounds are important performance indicators of the quality and efficiency of the interaction. In terms of negotiation success rate, our model achieves 100% with both the literature [6] and the literature [7] for the given dataset, which is significantly better than the literature [5]. As for the negotiation rounds indicator, the average number of negotiation rounds for the fastest method of negotiation [6] is about 2, which indicates that the method completes the negotiation almost at the very beginning of the negotiation and compromises too fast. Obviously, this is unreasonable and does not correspond to the reality of business negotiations [67]. In addition, it has been shown that the average number of negotiation rounds in human-agent negotiations generally does not exceed 20 [70]. Therefore, combining the above results, only our proposed model and the literature [7] are consistent with the reality, and the negotiation efficiency of this paper is better than that of the literature [7]. To sum up, the proposed method in this paper outperforms the other methods compared in terms of negotiation rounds.

Joint utility and utility difference are important performance indicators for measuring negotiation results. Specifically, the utility difference primarily reflects fairness, which is crucial in impressing both parties and serves as a significant factor in achieving the final deal. In terms of utility, while the proposed method in this paper yields joint utility results that are somewhat inferior to those reported in the literature [5], the latter exhibits a large utility difference, which indicates low fairness between the negotiating parties. By comparison, although our method shows a slightly worse utility difference than the literature [7], it produces better joint utility results. Overall, the results are comparable.

In summary, the results of the comparison with other methods can effectively show that the proposed model can effectively improve the quality and efficiency of the interaction. Meanwhile, our proposed model also has good performance in improving the joint utility of negotiation and reducing the utility difference. The above findings can prove the validity and advancement of the proposed model in this paper.

### 5.7 A practical application of the proposed model

In this subsection, we provide a case study to describe an application example of the proposed method. The application example focuses on the negotiations about purchasing thermal coal between Kailuan Group International Logistics Co., Ltd. (the buyer and hereafter referred to as KL) and Qinhuangdao Mingwei Economic and Trade Co., Ltd. (the seller and hereafter referred to as QM). KL is a large enterprise mainly engaged in the road transportation industry, which has a long-term and stable demand for coal energy. QM belongs to the wholesale industry, and the main business scope includes wholesale coal, steel, and building materials product sales. KL ordered thermal coal from QM every few months, with each purchase involving approximately 15000 metric tons. Since the thermal coal market has become more volatile, they have to negotiate with each other to settle the price and the calorific value, an important quality indicator of the thermal coal. In general, a high caloric value implies a high quality of the thermal coal. Previously both companies dispatched negotiating teams to engage in the human-led negotiations and it often took one or two days to arrive at the consensus. We contacted KL and introduced our developed agent-based automated negotiation system. KL showed interests in the negotiation system and agreed to try the system.

**Fig 8:**
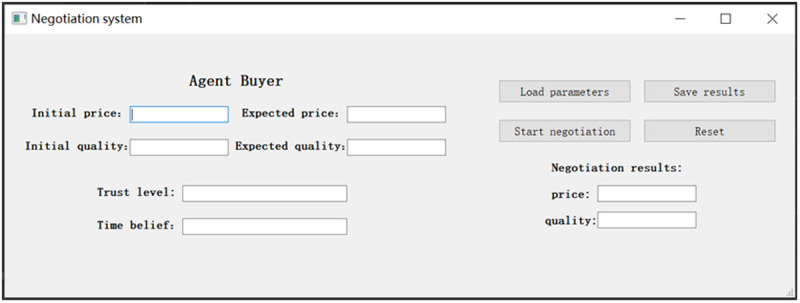
Main interface of the developed automated negotiation system.

The negotiation system was developed based on the proposed scheme. Fig 8 shows the operating screen. The left side of the screen contains the initial parameters needed to be provided by the user, including the first-round proposal values of price and quality, the expected transaction value of price and quality, the relationship between the negotiating parties (trust level), and the time attitude towards the negotiation (time belief). There are four buttons in the upper right of the screen, which are respectively for loading parameters, starting negotiation, saving results, and resetting. By clicking the button “Load parameters”, the system can obtain the initial parameter manually entered on the left side of the interface; click “Start negotiation”, and the system starts the automated negotiation program to interact with the opponent. The real-time negotiation results will be synchronously displayed at the lower right. When the negotiation is over, clicking “Save results” can save the negotiation results record in the system, and then clicking the button “Reset” can reset the system.

**Fig 9:**
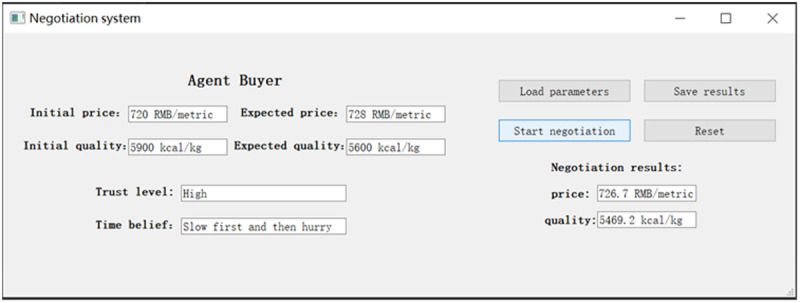
Results of automated negotiation system application.

KL set the initial price and the quality to be 720 RMB/metric ton and 5900 kcal/kg, respectively, and the expected transaction values of both attributes were set to be 728 RMB/metric ton and 5600 kcal/kg, respectively. These values are set according to the mean values of the latest five historical negotiations of LK and the current spot market of the thermal coal of the same specifications. KL and QM have a long-term cooperation and thus the “trust level” was set to be high. Meanwhile, KL preferred the slow-first-and-then-fast negotiation style and accordingly the “time belief” was set to be “slow first and fast later”. The negotiation results can be obtained by clicking the “Load parameters” and “Start negotiation” buttons successively, as shown in Fig 9.

**Fig 10:**
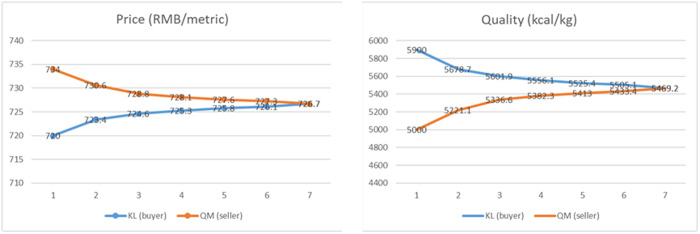
The negotiation process between KL and QM.

After seven rounds of negotiation, which took about 32 minutes, the two parties reached an agreement on the negotiating attributes. The counter-offer values for each round of negotiation between KL and QM are shown in Fig 10.

**Fig 11:**
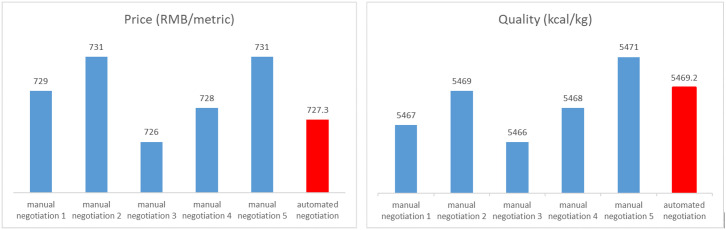
Comparison of the results between the manual negotiation and the proposed method.

In addition, we compare the results of five historical manual negotiations of KL with those generated by this automated negotiation system. The results are shown in Fig 11, where blue represents the results of the last five manual negotiations of KL and red represents the results of using the automated negotiation system.

By observing Fig 11, it can be seen that the method proposed in this paper can obtain a result comparable to that of manual negotiation. It’s worth mentioning that the automated negotiation results were both second only to the best record of manual negotiation in terms of price and quality. That is to say, high-quality thermal coal can be bought at a relatively low price with the automated negotiation system. Therefore, KL was satisfied with the automated negotiation results and expressed its willingness to cooperate with us to further revise the system.

## 6. Discussion

### 6.1 Theoretical implications

Herein, an emotion-driven model for automated negotiation is proposed, which integrates the dimensions of certainty and pleasantness into the selection of emotional persuasion strategies based on the ATF. The concession update algorithm is improved by incorporating a time function, which increases the intelligence of the persuasion process. A series of numerical experiments evaluated the effectiveness of the proposed model with the mentioned theoretical implications.

The key innovation of the proposed model lies in dynamically selecting emotional persuasion strategies based on the ATF to enhance agent-based negotiation with opponents. The ATF, a well-established theory concerning the influence of specific emotions on consumer perceptions and decision making, has been widely used in areas such as risk assessment and value evaluation. However, to the best of our knowledge, this is the first study to integrate the ATF in agent-based automated negotiation. This study focused on leveraging the ATF to draw causal inferences, improving the interpretability of the negotiation process, from cognitive evaluation modeling to emotional strategy selection. It is expected to bridge the gap between the theoretical and practical applications of the ATF for diverse applications.

This study identifies “realism” as the primary goal of emotion modeling—preserving and leveraging the full spectrum of emotions and their corresponding cognitive appraisal tendencies throughout multi-round negotiations, thereby enabling agents to develop process-oriented regulation mechanisms similar to those of humans. “Effectiveness” refers to the natural performance that emerges under varying opponent types, bargaining intensities, and time pressures. Compared with agents that adopt only a positive attitude, the complete emotional expression, mapped through the cognitive dimensions of the ATF, offers a broader strategic space and greater adaptability.

Human emotions possess motivational properties that enable individuals to dynamically adjust their judgments and decisions, independent of past experiences. This paper integrates human emotions to enhance the automation and flexibility of agent-based systems in selecting appropriate emotional persuasion strategies within dynamic environments. While numerous studies have explored modeling agents’ emotions, most existing literature focuses on simulating the generation of emotions and their direct influence on decision-making. In contrast, the current study differentiates itself by deconstructing emotions into cognitive dimensions, which are then mapped automatically onto emotional persuasion strategy selection processes using a flexible yet specific framework: the Appraisal Tendency Framework. The results of the ablation study further validate the effectiveness of this approach. This study validates the effectiveness of emotional persuasion strategies and time-based concession mechanisms in an automated–automated negotiation scenario, demonstrating that their combination can significantly improve negotiation success rate, joint utility, and fairness. It is worth noting that although the experimental setting of this study is limited to automated–automated negotiations, the proposed emotional appraisal mechanism and time-based concession modeling framework are not confined to such scenarios. Theoretically, this framework is equally applicable to human–agent negotiation contexts. In future work, we will conduct human–agent experiments based on the existing model, incorporating interaction log analysis between humans and machines while keeping the core mechanisms unchanged, in order to assess the framework’s applicability and value in human–agent environments.

The time-belief function represents a negotiator’s attitude toward time. In this paper, we propose two kinds of time belief functions with a non-constant rate of change, reflecting the dynamic nature of agents’ attitudes toward time pressure. This approach offers more practical options for the agent, depending on the urgency of time. Meanwhile, this study explores how varying time parameters affect negotiation results through extensive comparative experiments. The experimental findings indicate that the time-belief functions introduced in this work significantly enhance the effectiveness of emotional persuasion, outperforming other benchmark methods. Furthermore, we analyze the specific influence of different time-belief parameter configurations on concessions and present corresponding conclusions in Section 5. Overall, this research provides some valuable insights for future studies in selecting or designing appropriate time functions.

### 6.2 Practical implications

This study has significant potential for a wide range of practical applications in the fields of automated negotiation and intelligent decision-making within business intelligence. It is well established that the design of an agent’s capability framework fundamentally determines the level of intelligence it can exhibit, which in turn forms the foundation for constructing an intelligent interactive system. From the perspective of negotiation system design, the proposed model can be viewed as an extension of traditional automated negotiation systems, as it incorporates not only human emotions but also responses to time pressure. Specifically, the novel automated negotiation system proposed in this paper addresses user expectations for machines that exhibit anthropomorphic characteristics, while also accommodating the diverse time-related preferences of different users.

The system is structured as follows: the core emotion module consists of two sub-modules designed to model (1) the level of trust between negotiating partners, and (2) the degree of pleasantness experienced during the real-time negotiation process. This basic emotion module is flexible and can be expanded to incorporate additional cognitive dimensions as outlined in the Appraisal Tendency Framework theory. For instance, the responsibility dimension could be modeled based on the negotiation partner’s historical performance. The second component of the system is the time module, which features adaptive matching capabilities. This module can generate different time belief functions tailored to the negotiators’ time attitudes. For the purposes of this paper, two temporal attitudes are initially proposed for selection. However, this module can also be further extended based on the time-belief functions discussed herein, allowing for additional customization according to specific system requirements, thereby offering more options to the negotiating parties. In summary, by developing and extending the emotional persuasion model presented in this paper, it is possible to create a more practical and effective artificial intelligence-based automated negotiation system.

This positioning also provides a methodological cornerstone for extending to human–machine negotiation. Under interaction conditions where human emotions are more uncertain and volatile, complete emotional expression and process-oriented regulation become particularly critical. In future work, we will explore context-aware emotional adaptive selection to achieve a better balance among realism, effectiveness, and efficiency.

Moreover, a comprehensive understanding of how to effectively use the negotiation system is essential for its successful implementation. Managers should assess which model offers the greatest practical benefits by accurately evaluating the company’s specific conditions and needs. The proposed model is parameter-rich, providing greater flexibility and enabling companies to adjust parameters according to their unique circumstances. For example, in a profit-driven organization, if the company has a high level of trust, negotiating with a party that has a significantly lower trust level may yield greater benefits. Conversely, if the company’s trust level is low, it may be more advantageous to negotiate with a party whose trust level is closer to its own. Similarly, the choice of time-belief function depends on the company’s strategic priorities. If the company places a high value on negotiation efficiency and the total utility of both parties, the time-belief function F2 should be selected. However, if the primary concern is the utility difference between the parties, the time-belief function F1 or another fixed-speed time-belief function would be more appropriate. In summary, by accurately identifying the company’s needs and strategically utilizing the automated negotiation system, greater value can be created for the organization.

While it is acknowledged that human negotiators often possess superior negotiation skills, which can lead to more favorable outcomes, the human and time costs associated with human-to-human negotiations are significantly higher than those involved in human-to-machine negotiations. This is especially evident in B2C e-commerce environments, where a large number of buyers are involved. Consequently, we are confident that the proposed automated negotiation system can serve as a valuable complement to human-human negotiations in online e-commerce settings. In addition to B2C e-commerce platforms, the potential applications of this negotiation system extend beyond these environments and may include, but are not limited to, enterprise procurement processes and carbon emission trading systems, etc.

### 6.3 Limitations

The limitations of this study are mentioned below.

(1) While the proposed model emphasizes the role of cognitive appraisal and develops a method for selecting emotional persuasion strategies based on the ATF, it considers two primary cognitive dimensions. Future research can expand the model by incorporating additional dimensions, such as attentional engagement and responsibility, to comprehensively capture the personalized characteristics of agents. Regarding emotional strategy selection, it primarily establishes discrete correspondences. Future studies can explore the internalization of these strategies within cognitive dimensions, potentially establishing continuous, linear, or nonlinear relationships.
(2) Owing to the confidential nature of commercial negotiation data, which is not readily accessible, this model primarily relied on numerical simulations for validation. Future studies can incorporate laboratory experiments by collecting empirical data to further test and refine the validity of the model.
(3) Although the current model considers both emotional and temporal factors, they are modeled independently, without reflecting the dynamic regulatory effect of emotions on time perception. Future work can introduce coupling parameters to map emotional dimensions to the parameters of the time function, allowing time pressure perception to vary with emotional fluctuations. Controlled experiments can then be designed to compare negotiation outcomes under independent and coupled modeling, in order to verify performance differences and applicability.

## 7. Conclusion

This study enhanced the modeling of agent-based persuasion behavior in automated negotiation by emphasizing the roles of emotion and timing. The concession process of the model was developed within the framework of emotional persuasion based on the ATF, focusing on two cognitive dimensions: certainty and pleasantness. Additionally, the proposed concession update algorithm was improved by incorporating a time function, enabling the agent to consider the urgency of time and emotional persuasion in making concessions. The results demonstrated that integrating emotion and timing into automated negotiation significantly improved the performance. Our proposed model outperformed existing persuasion models in terms of persuasion SR, negotiation efficiency, and overall social welfare. Consequently, this study provided valuable insights for the design of advanced automated negotiation systems.

## References

[1] Gao T-G, Huang M, Wang Q, Wang X-W. Dynamic organization model of automated negotiation for 3PL providers selection. Information Sciences. 2020;531:139–58. doi: 10.1016/j.ins.2020.03.086

[2] Eshragh F, Shahbazi M, Far B. Real-time opponent learning in automated negotiation using recursive Bayesian filtering. Expert Systems with Applications. 2019;128:28–53. doi: 10.1016/j.eswa.2019.03.025

[3] Lee SK, Kavya P, Lasser SC. Social interactions and relationships with an intelligent virtual agent. International Journal of Human-Computer Studies. 2021;150:102608. doi: 10.1016/j.ijhcs.2021.102608

[4] Wei Y, Lu W, Cheng Q, Jiang T, Liu S. How humans obtain information from AI: Categorizing user messages in human-AI collaborative conversations. Information Processing & Management. 2022;59(2):102838. doi: 10.1016/j.ipm.2021.102838

[5] Cao M, Hu Q, Kiang MY, Hong H. A Portfolio Strategy Design for Human-Computer Negotiations in e-Retail. International Journal of Electronic Commerce. 2020;24(3):305–37. doi: 10.1080/10864415.2020.1767428

[6] Keskin MO, Çakan U, Aydoğan R. Solver agent: towards emotional and opponent-aware agent for human-robot negotiation. In: Proceedings of the 20th International Conference on Autonomous Agents and MultiAgent Systems, 2021.

[7] Wu J, Zhang T, Li Y, Zhou G. Emotion-driven reasoning model for agent-based human–computer negotiation. Expert Systems with Applications. 2024;240:122448. doi: 10.1016/j.eswa.2023.122448

[8] Schweitzer F, Krivachy T, Garcia D. An Agent-Based Model of Opinion Polarization Driven by Emotions. Complexity. 2020;2020:1–11. doi: 10.1155/2020/5282035

[9] Wang Z, Shen J, Tang X, Han M, Feng Z, Wu J. An agent-based persuasion model using emotion-driven concession and multi-objective optimization. Auton Agent Multi-Agent Syst. 2024;38(2). doi: 10.1007/s10458-024-09664-7

[10] Carnevale PJ. Strategic time in negotiation. Curr Opin Psychol. 2019;26:106–12. doi: 10.1016/j.copsyc.2018.12.017 30718224

[11] Carr A. It’s about time: Strategy and temporal phenomena. Journal of Strategic Studies. 2018;44(3):303–24. doi: 10.1080/01402390.2018.1529569

[12] Ordóñez LD, Benson L III, Pittarello A. Time‐pressure Perception and Decision Making. The Wiley Blackwell Handbook of Judgment and Decision Making. Wiley. 2015. 517–42. doi: 10.1002/9781118468333.ch18

[13] Rodríguez L-F, Ramos F. Development of Computational Models of Emotions for Autonomous Agents: A Review. Cogn Comput. 2014;6(3):351–75. doi: 10.1007/s12559-013-9244-x

[14] Ruijten PAM, Midden CJH, Ham J. Ambiguous Agents: The Influence of Consistency of an Artificial Agent’s Social Cues on Emotion Recognition, Recall, and Persuasiveness. International Journal of Human–Computer Interaction. 2016;32(9):734–44. doi: 10.1080/10447318.2016.1193350

[15] Yang J, Li Z, Du X. Analyzing audiovisual data for understanding user’s emotion in human−computer interaction environment. DTA. 2023;58(2):318–43. doi: 10.1108/dta-08-2023-0414

[16] Wu J, Zhang Y, Cao R, Li Y. An agent-based emotional persuasion model driven by integrated trust assessment. Engineering Applications of Artificial Intelligence. 2025;149:110567. doi: 10.1016/j.engappai.2025.110567

[17] Ramchurn S. Multi-Agent Negotiation using Trust and Persuasion. University of Southampton. 2004.

[18] Ochsner KN, Gross JJ. The cognitive control of emotion. Trends Cogn Sci. 2005;9(5):242–9. doi: 10.1016/j.tics.2005.03.010 15866151

[19] Martinovski B, Mao W. Emotion as an Argumentation Engine: Modeling the Role of Emotion in Negotiation. Group Decis Negot. 2009;18(3):235–59. doi: 10.1007/s10726-008-9153-7

[20] Wu J, Zhang F, Han J, Li Y, Sun Y. Agent-based automated persuasion with adaptive concessions tuned by emotions. J Ambient Intell Humaniz Comput. 2022;13(6):2921–35. doi: 10.1007/s12652-021-03089-w 33758627 PMC7971364

[21] Xiao X, Mao W, Sun Y, Zeng D. A cognitive emotion model enhanced sequential method for social emotion cause identification. Information Processing & Management. 2023;60(3):103305. doi: 10.1016/j.ipm.2023.103305

[22] Liu F, Wang H-Y, Shen S-Y, Jia X, Hu J-Y, Zhang J-H, et al. OPO-FCM: A Computational Affection Based OCC-PAD-OCEAN Federation Cognitive Modeling Approach. IEEE Trans Comput Soc Syst. 2023;10(4):1813–25. doi: 10.1109/tcss.2022.3199119

[23] Rasmussen AS, Berntsen D. Emotional valence and the functions. Memory & Cognition. 2009;37:477–92. 19460954 10.3758/MC.37.4.477

[24] Lerner JS, Keltner D. Fear, anger, and risk. J Pers Soc Psychol. 2001;81(1):146–59. doi: 10.1037//0022-3514.81.1.146 11474720

[25] Zeelenberg M, Pieters R. Beyond valence in customer dissatisfaction: A review and new findings on behavioral responses to regret and disappointment in failed services. Journal of Business Research. 2004;57(4):445–55.

[26] Han S, Lerner JS, Keltner D. Feelings and Consumer Decision Making: The Appraisal‐Tendency Framework. J Consum Psychol. 2007;17(3):158–68. doi: 10.1016/s1057-7408(07)70023-2

[27] Smith CA, Ellsworth PC. Patterns of cognitive appraisal in emotion. Journal of Personality and Social Psychology. 1985;48(4):813–38. doi: 10.1037/0022-3514.48.4.813 3886875

[28] Pathak S, Srivastava KB, Dewangan RL. An Experimental Comparison of Two Emotion‐Induction Methods and the Role of Emotion in Applying Decision Rules. Jpn Psychol Res. 2023. doi: 10.1111/jpr.12466

[29] Kolomvatsos K, Trivizakis D, Hadjiefthymiades S. An adaptive fuzzy logic system for automated negotiations. Fuzzy Sets and Systems. 2015;269:135–52. doi: 10.1016/j.fss.2014.09.016

[30] Imran K, Zhang J, Pal A, Khattak A, Ullah K, Baig SM. Bilateral negotiations for electricity market by adaptive agent-tracking strategy. Electric Power Systems Research. 2020;186:106390. doi: 10.1016/j.epsr.2020.106390

[31] Bagga P, Paoletti N, Alrayes B, Stathis K. ANEGMA: an automated negotiation model for e-markets. Auton Agent Multi-Agent Syst. 2021;35(2). doi: 10.1007/s10458-021-09513-x

[32] Zhang Y, Wu J, Cao R. Optimizing Automated Negotiation: Integrating Opponent Modeling with Reinforcement Learning for Strategy Enhancement. Mathematics. 2025;13(4):679. doi: 10.3390/math13040679

[33] Aydogan R, Festen D, Hindriks KV, Jonker CM, editors. Alternating Offers Protocols for Multilateral Negotiation. Workshop on modern approaches to agent-based complex automated negotiation; 2017.

[34] Jennings NR, Faratin P, Lomuscio AR, Parsons S, Wooldridge MJ, Sierra C. Automated Negotiation: Prospects, Methods and Challenges. Group Decision & Negotiation. 2001;10(2):199–215.

[35] Hausken K. Game-theoretic and behavioral negotiation theory. Group Decision and Negotiation. 1997;6:511–28.

[36] Rahwan I, Ramchurn SD, Jennings NR, Mcburney P, Parsons S, Sonenberg L. Argumentation-based negotiation. The Knowledge Engineering Review. 2003;18(4):343–75. doi: 10.1017/s0269888904000098

[37] Rahwan I, Sonenberg L, Jennings NR, McBurney P. Stratum: A methodology for designing heuristic agent negotiation strategies. Applied Artificial Intelligence. 2007;21(6):489–527.

[38] Wade J. Persuasion in Negotiation and Mediation. Law and Justice Journal. 2008;8(1):253–78.

[39] Ghalayini L, Deeb D. Utility measurement in integrative negotiation. Information Management and Business Review. 2021;13(1):1–15.

[40] Lai G, Li C, Sycara K. Efficient multi-attribute negotiation with incomplete information. Group Decision and Negotiation. 2006;15:511–28.

[41] Barry B, Fulmer IS, Kleef GV. I laughed, I cried, I settled: The role of emotion in negotiation. Spine. 2004;57(3):71-–94. doi: 10.1007/s11528-013-0657-x

[42] Raghunathan R, Pham M. All Negative Moods Are Not Equal: Motivational Influences of Anxiety and Sadness on Decision Making. Organ Behav Hum Decis Process. 1999;79(1):56–77. doi: 10.1006/obhd.1999.2838 10388609

[43] van Kleef GA, De Dreu CKW, Manstead ASR. The interpersonal effects of anger and happiness in negotiations. J Pers Soc Psychol. 2004;86(1):57–76. doi: 10.1037/0022-3514.86.1.57 14717628

[44] Chong DSF, Eerde WV, Chai KH, Rutte CG. A Double-Edged Sword: The Effects of Challenge and Hindrance Time Pressure on New Product Development Teams. IEEE Trans Eng Manage. 2011;58(1):71–86. doi: 10.1109/tem.2010.2048914

[45] Wu J, Chen H, Li Y, Liu Y. A Behavioral Assessment Model for Emotional Persuasion Driven by Agent-Based Decision-Making. Expert Systems with Applications. 2022;204:117556. doi: 10.1016/j.eswa.2022.117556

[46] Pinfari M. Time to Agree. Journal of Conflict Resolution. 2011;55(5):683–709. doi: 10.1177/0022002711414370

[47] Evans P, Vansteenkiste M, Parker P, Kingsford-Smith A, Zhou S. Cognitive Load Theory and Its Relationships with Motivation: a Self-Determination Theory Perspective. Educ Psychol Rev. 2024;36(1). doi: 10.1007/s10648-023-09841-2

[48] Bellantuono N, Kersten GE, Pontrandolfo P. Markets of Logistics Services: The Role of Actors’ Behavior to Enhance Performance. Behavioral Issues in Operations Management. Springer London. 2013. p. 221–36. doi: 10.1007/978-1-4471-4878-4_11

[49] Carbonneau R, Vahidov R. A Multi-attribute bidding strategy for a single-attribute auction marketplace. Expert Systems with Applications. 2016;43:42–50. doi: 10.1016/j.eswa.2015.08.039

[50] Yao J, Storme M. Trust Building via Negotiation: Immediate versus Lingering Effects of General Trust and Negotiator Satisfaction. Group Decis Negot. 2021;30(3):507–28. doi: 10.1007/s10726-020-09721-y

[51] Basheer GS, Ahmad MS, Tang AYC, Graf S. Certainty, trust and evidence: Towards an integrative model of confidence in multi-agent systems. Computers in Human Behavior. 2015;45:307–15. doi: 10.1016/j.chb.2014.12.030

[52] Nguyen TD, Bai Q. A Dynamic Bayesian Network approach for agent group trust evaluation. Computers in Human Behavior. 2018;89:237–45. doi: 10.1016/j.chb.2018.07.028

[53] Fan S. A Multiagent Cooperation Model Based on Trust Rating in Dynamic Infinite Interaction Environment. Mathematical Problems in Engineering. 2018;2018:1–11. doi: 10.1155/2018/2089596

[54] Roseman IJ, Smith CA. Appraisal theory. Appraisal processes in emotion: Theory, methods, research. 2001:3-–19. 10.1002/0470013494.ch30

[55] Nutter FW Jr, Esker PD. The Role of Psychophysics in Phytopathology: The Weber–Fechner Law Revisited. Eur J Plant Pathol. 2006;114(2):199–213. doi: 10.1007/s10658-005-4732-9

[56] Ekman P. Basic emotions. Handbook of cognition and emotion. 1999. p. 45–60.

[57] Kraus S, Sycara K, Evenchik A. Reaching agreements through argumentation: a logical model and implementation. Artificial Intelligence. 1998;104(1–2):1–69. doi: 10.1016/s0004-3702(98)00078-2

[58] Ekman P. An argument for basic emotions. Cognition and Emotion. 1992;6(3–4):169–200. doi: 10.1080/02699939208411068

[59] Santos R, Marreiros G, Ramos C, Neves J, Bulas-Cruz J. Personality, Emotion, and Mood in Agent-Based Group Decision Making. IEEE Intell Syst. 2011;26(6):58–66. doi: 10.1109/mis.2011.92

[60] Kapucu A, Rotello CM, Yüvrük E. Recognition memory for specific emotion words: anger, fear, and disgust. Motiv Emot. 2024;48(5):791–806. doi: 10.1007/s11031-024-10084-z

[61] Lerner JS, Keltner D. Beyond valence: Toward a model of emotion-specific influences on judgement and choice. Cogn Emot. 2000;14(4):473–93.

[62] Chua HF, Gonzalez R, Taylor SF, Welsh RC, Liberzon I. Decision-related loss: regret and disappointment. Neuroimage. 2009;47(4):2031–40. doi: 10.1016/j.neuroimage.2009.06.006 19524050

[63] Butler RM, Boden MT, Olino TM, Morrison AS, Goldin PR, Gross JJ, et al. Emotional clarity and attention to emotions in cognitive behavioral group therapy and mindfulness-based stress reduction for social anxiety disorder. J Anxiety Disord. 2018;55:31–8. doi: 10.1016/j.janxdis.2018.03.003 29558650 PMC5879018

[64] Moore DJ, Lee SP. How advertising influences consumption impulses the role of visualization, anticipated emotions, taste anticipation, and hedonic rationalization. Journal of Advertising. 2012;41(3):107-–20. doi: 10.2753/joa0091-3367410307

[65] Antonetti P, Baines P, Jain S. The persuasiveness of guilt appeals over time: Pathways to delayed compliance. Journal of Business Research. 2018;90:14–25. doi: 10.1016/j.jbusres.2018.03.030

[66] Xie X, Liu L. Exploring the antecedents of trust in electronic word-of-mouth platform: The perspective on gratification and positive emotion. Front Psychol. 2022;13:953232. doi: 10.3389/fpsyg.2022.953232 36059728 PMC9434003

[67] Chen L, Dong H, Zhou Y. A reinforcement learning optimized negotiation method based on mediator agent. Expert Systems with Applications. 2014;41(16):7630–40. doi: 10.1016/j.eswa.2014.06.003

[68] Ros R, Sierra C. A Negotiation Meta Strategy Combining Trade-off and Concession Moves. Auton Agent Multi-Agent Syst. 2006;12(2):163–81. doi: 10.1007/s10458-006-5837-z

[69] Lee C-F, Chang P-L. Evaluations of Tactics for Automated Negotiations. Group Decis Negot. 2008;17(6):515–39. doi: 10.1007/s10726-008-9109-y

[70] Peled N, Gal Y, Kraus S. A study of computational and human strategies in revelation games. Auton Agent Multi-Agent Syst. 2014;29(1):73–97. doi: 10.1007/s10458-014-9253-5

